# Clinical detection, diagnosis and treatment of morphological abnormalities of sperm flagella: A review of literature

**DOI:** 10.3389/fgene.2022.1034951

**Published:** 2022-11-08

**Authors:** Jiaxiong Wang, Weizhuo Wang, Liyan Shen, Aiyan Zheng, Qingxia Meng, Hong Li, Shenmin Yang

**Affiliations:** Center for Reproduction and Genetics, Suzhou Municipal Hospital, The Affiliated Suzhou Hospital of Nanjing Medical University, Gusu School, Nanjing Medical University, Suzhou, China

**Keywords:** MMAF, intracytoplasmic sperm injection, clinical detection, diagnosis, genetic

## Abstract

Sperm carries male genetic information, and flagella help move the sperm to reach oocytes. When the ultrastructure of the flagella is abnormal, the sperm is unable to reach the oocyte and achieve insemination. Multiple morphological abnormalities of sperm flagella (MMAF) is a relatively rare idiopathic condition that is mainly characterized by multiple defects in sperm flagella. In the last decade, with the development of high-throughput DNA sequencing approaches, many genes have been revealed to be related to MMAF. However, the differences in sperm phenotypes and reproductive outcomes in many cases are attributed to different pathogenic genes or different pathogenic mutations in the same gene. Here, we will review information about the various phenotypes resulting from different pathogenic genes, including sperm ultrastructure and encoding proteins with their location and functions as well as assisted reproductive technology (ART) outcomes. We will share our clinical detection and diagnosis experience to provide additional clinical views and broaden the understanding of this disease.

## Introduction

Approximately 15% of couples suffer from infertility worldwide, and in half of those cases the disease is caused by male factors (Agarwal et al., 2015; Minhas et al., 2021). Sperm concentration, motility, morphology and DNA integrity greatly affect male fertility potential. The fertility potential of the sperm is so fragile that many factors have impact on it, such as male age, living habits, environmental factors, chemical exposure or radiation exposure. Among all the influencing factors, genetic defects, although only a small part, attract attention due to the uniform and severe phenotypes, and many cases with severe astheno- and/or teratozoospermia are related to genetic defects.

Multiple morphological abnormalities of sperm flagella (MMAF) is a type of severe asthenoteratozospermia characterized by a series of flagella anomalies, such as absent, short, bent or coiled flagella and flagella of irregular caliber (Wang W. et al., 2020). The first gene reported to be related to MMAF was *DNAH1* (Ben Khelifa et al., 2014), and with the development of high-throughput DNA sequencing approaches, additional pathogenic genes have been revealed and are discussed below in detail.

Due to severely reduced sperm motility, the most appropriate and only assisted reproductive technology for MMAF patients is intracytoplasmic sperm injection (ICSI). Most MMAF patients obtain a favorable ICSI outcome, but failed cases still occur, which may be attributed to various protein functional defects caused by different gene variants, some of which may affect embryonic development.

In recent years, most researchers have focused on the discovery of MMAF pathogenic genes, and a summary of MMAF clinical diagnosis and treatment is lacking. We have previously published diagnosis and treatment options for MMAF in some Chinese journals (Yang et al., 2018) but with no systematic summary. Here, we summarized our experience of MMAF diagnosis and treatment over the decade and reviewed the different phenotypes and ICSI outcomes caused by various pathogenic genes and mutations as well as provided a more comprehensive reference for the clinical diagnosis and treatment of MMAF.

### Sperm flagella structure

**FIGURE 1:**
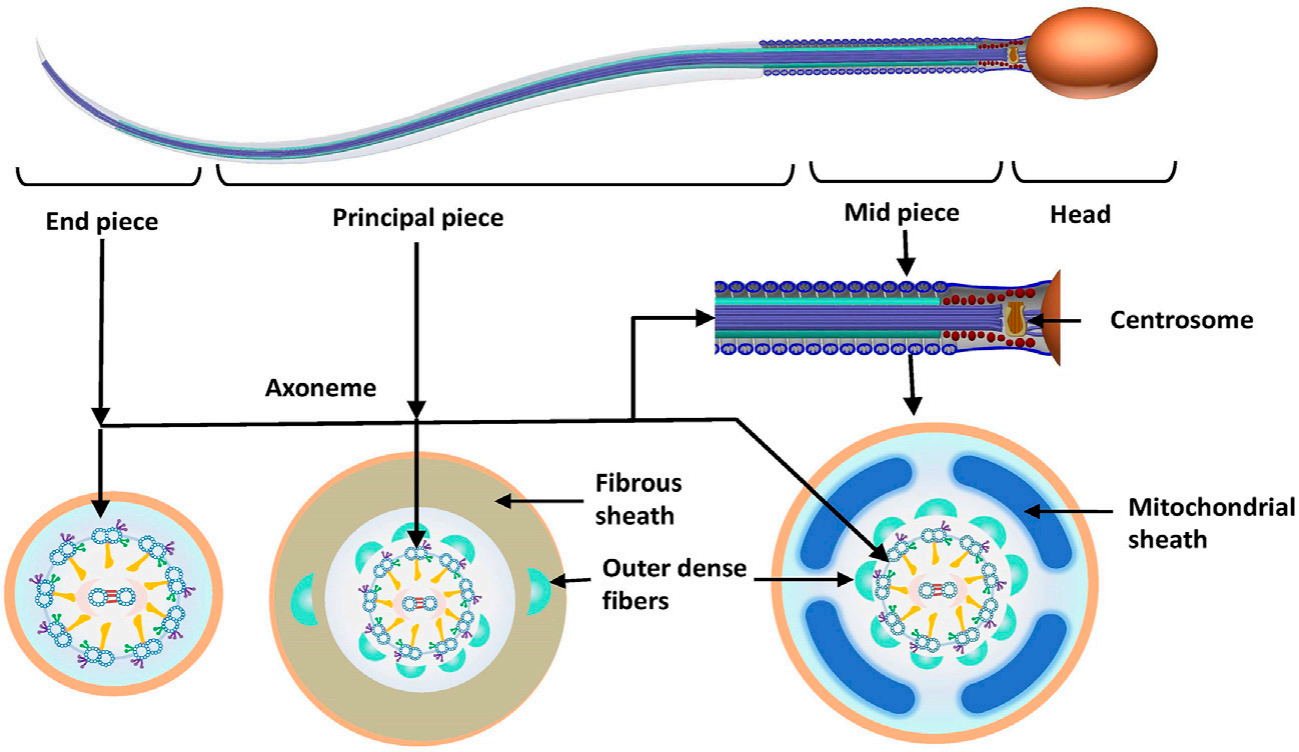
Normal sperm morphology and ultrastructure. Sperm is divided into the sperm head and flagellum. The flagellum is subdivided into the following three parts: midpiece, principal piece and end piece. The centrosome is divided into proximal and distal centrioles, which attach to the basal plate at the implantation fossa and form the axoneme. The axoneme crosses through the entire flagellum. In the midpiece and principal piece, the axoneme is surrounded by outer dense fibers. The midpiece also consists of a mitochondrial sheath surrounding the axoneme, which is replaced by the fibrous sheath in the principal piece. There is no periaxonemal structure in the end piece.

To better understand the pathogenic mechanism of MMAF, it is necessary to determine sperm morphology and the ultrastructure of sperm flagella. Sperm can be divided into a head and a tail or a flagellum, which are joined by the connecting piece (neck). The flagellum can be further subdivided into midpiece, principal piece and endpiece. The length of the sperm flagellum is 10 times longer than the head (approximately 55 μm), which comprises a series of well-structured components to ensure that it provides enough power to propel the sperm to swim. The axoneme, which crosses the entire length of the sperm, consists of the central support structure of the flagellum. The core structure of axoneme, the “9 + 2” structure, consists of a central microtubule pair surrounded by nine peripheral doublet microtubules linked by nexin, which are connected through radial spokes (RSs). Each pair of double microtubules is divided into A and B microtubules. Between each set of A and B microtubules are two dynein arms called the inner and outer dynein arms (IDAs and ODAs, respectively). The dynein arms are projected from microtubule A of the previous doublet toward microtubule B of the next doublet. In the midpiece, the axoneme is enclosed by the segmented columns. Distal ends of the segmented columns attach to the outer dense fibers (ODFs), which surround the peripheral doublet microtubules, and the position corresponds to them. The mitochondrial sheath (MS) is formed with the accumulation of mitochondria around the ODFs in the midpiece. In the principal piece, ODF3 and ODF8 are fused with the lateral columns of the fibrous sheath (FS) (Figure 1).

### Recognition of the MMAF phenotype

The understanding of the sperm phenotype of MMAF patients has increased with the development of microscopic observation technology. The use of a light microscope by Antonie van Leeuwenhoeke to observe semen and the invention of Papanicolaou staining have allowed sperm morphology to be observed and applied to clinical detection (Wang J. et al., 2022). Subsequently, the criteria for the classification of sperm morphology have been continuously improved, and the strict criteria of the World Health Organization (WHO) laboratory manual for the examination and processing of human semen has been established. Constant developments and innovations in observation technology are indicated by the evolution of the name of this disease. Initially, due to the widespread use of light microscopy, this type of disease was called short tail or stump tail sperm, reflecting only the most intuitive shape defect of sperm in these patients (Terquem and Dadoune, 1980). With the invention of the electronic microscope, the ultrastructure of sperm flagella was revealed. Chemes et al. termed this disease as DFS for the first time. In their study, the major flagellar ultrastructure defect observed in the sperm of five patients was hyperplastic and disorganized fibrous sheath (Chemes et al., 1987). With an increasing number of studies, researchers have found that this nomenclature is one-sided because the ultrastructural defects of the sperm flagella of the patients vary even if they appear similar in shape under the light microscope. Therefore, some scholars have suggested that this syndrome should be called multiple morphological abnormalities of sperm flagella (MMAF), which has been widely used and presented in the sixth edition of the WHO laboratory manual for the examination and processing of human semen (Coutton et al., 2015; Björndahl et al., 2021). However, several researchers still insist that DFS and MMAF are two distinct syndromes, because unlike MMAF, the main ultrastructural defect of DFS is in FS, which is noteworthy (Sha Y. et al., 2017; Oud et al., 2021). As mentioned above, sperm flagella are comprised of many submicroscopic structures. Although MMAF sperm flagella under the light microscope can be classified into one category according to the proportion of different types of flagella defects, the inner ultrastructure defects maybe different due to various protein dysfunctions resulting different gene defects. Therefore, we speculated that MMAF should be subdivided into subtypes based on subtle differences in its flagellar ultrastructural defects and pathogenic genes.

## Clinical diagnosis of MMAF

At present, the clinical examination of MMAF patients involves three factors. Routine clinical examination is one of them. In general, MMAF patients show no defects upon physical examination and no specific clinical symptoms, but some present with varicocele. Most of the MMAF cases inherit in an autosomal recessive pattern, indicating that MMAF often occurs in consanguineous families. When a patient shows abnormal sperm motility and severe abnormal flagella morphology as well as has consanguineous parents, there is an increased possibility of MMAF. When the patient comes to the clinic for consultation, medical history collection is important, including the patient’s reproductive history and exposure history to tobacco, alcohol, high temperature, chemicals, radiation, special drugs and poisons**.** The patient’s medical history should be recorded in detail, including chronic respiratory disease, fever, mumps, epididymitis, urogenital diseases and systemic diseases. The marital history and health status of the patient’s parents, siblings and grandparents should be recorded. As routine indicators of sperm quality, sperm concentration, motility and morphology may provide a preliminary reference. For MMAF patients, the motile sperm percentage is extremely low, even zero in almost half of the cases. Under a light microscope, sperm flagella show multiple abnormalities (absent, short, coiled, bent and irregular caliber flagella) and are recorded as the proportion of different types of flagella defects. In addition to flagella, some MMAF patients also have severe abnormalities in the sperm head and extremely low sperm concentration, which are attributed to the role of their pathogenic genes in spermatogenesis (Wang W. et al., 2021). Due to low motility, several tests, including sperm viability, sex hormones, reproductive system ultrasound, karyotype analysis and Y chromosome mircodeletion, are useful for differential diagnosis (Yang et al., 2018). In most cases, the results of these tests are all normal. Although there have been several studies reporting the relationship between sperm chromosomal and flagellar abnormalities, attributing the phenomenon to defects in the common components shared among the sperm centrosome, mitotic spindle and flagella (Baccetti et al., 2005a; Ghedir et al., 2014), chromosomal abnormalities are rarely detected in the genomes of MMAF patients. Clinically, many doctors hope to have a reliable cutoff value for sperm motility and morphological defects for distinguishing MMAF patients from other asthenozoospermia. In most cases, the sperm motility percentages of MMAF patients are 0%–10%, but there are still reports of motility greater than 10% (Wang W. L. et al., 2020). Therefore, 10% should be used as a reference, but it is not absolute. Reference limits of the percentage of different flagellar defects has been proposed by Liu et al. (2021a) according to the distribution range of morphologically normal spermatozoa observed in 926 fertile individuals, and we used these limits in our subsequent case reports (Wang J. et al., 2021). However, it is still too early to use these limits for clinical diagnosis.

The second factor of the clinical examination of MMAF is ultrastructure observation. The collection process and pretreatment of samples are key procedures for the observation of flagella ultrastructure. The death and apoptosis of the sperm may lead to deconstruction of the flagella ultrastructure, which would interfere with diagnosis. A proper processing of sperm cells for ultrastructural studies to avoid any artifacts related with cell dead is very necessary. We propose the sperm centrifugation and rinsing treatments recored in the many previous literatures (Tang et al., 2017; Liu et al., 2019a), which can effectively remove cell debris and impurities. MMAF presents a heterogenous phenotype, in part due to high number of genes involved, but still characterized by altered flagellar core structures. The main ultrastructure defects of MMAF sperm are the defects in the axoneme (missing central microtubule pairs or disordered arrangement of peripheral microtubules) and missing IDA, ODA or both as well as disorganization or hyperplasia of the ODF and FS. The proportion of various ultrastructural abnormalities of MMAF caused by different pathogenic genes is different, but the axonemes are all obviously abnormal. This is different from primary ciliary dyskinesia (PCD), which is characterized mainly by the ODA defects, and partly by the microtubular disorganization and IDA defects or ODA–IDA defects (Wallmeier et al., 2020). Due to the high price and maintenance costs of electron microscopes, ultrastructure observation of sperm is still mainly used for scientific research rather than clinical testing, and only a few large reproductive centers in China have introduced this technology. However, with the increasing number of studies and the popularization of technology, ultrastructure observation may become a commonly used sperm morphology test in the clinic.

The third and most important factor of the clinical examination of MMAF is genetic analysis, including next generation sequencing technologies, Sanger sequencing and single nucleotide polymorphism (SNP) arrays. In common condition, SNP array is not needed. However, when uniparental disomy (UPD) occurs, it is very essential (Wang W. et al., 2021). In the recent decade, many pathogenic genes have been revealed due to the development of high-throughput DNA sequencing approaches. The preliminary preparation for testing also plays a key role in clinical genetic detection, especially the collection of pedigree samples, sperm specimens for post-validation and the accuracy of phenotypic descriptions. It is worth noting that the currently reported pathogenic genes and pathogenic mutations related to MMAF are only the tip of the iceberg because unexplained new mutations or even new genes often appear in the clinic. At this time, follow-up pedigree verification, gene expression verification and even animal experiments become important. Similar to ultrastructure observation, the cost of genetic analysis is relatively high compared to the routine fertility potential detection, and the unexplainable variants provide interference and reference at the same time. Nevertheless, genetic analysis is the key step in the diagnosis of MMAF patients.

Above all, more and more new technologies are being used to help comprehensively and accurately diagnose MMAF. When selecting a test to use, doctors should consider the effect and costs of the test based on the phenotype of the patient and provide a personalized laboratory diagnosis plan for each patient.

## Pathogenic genes and their function inspermatogenesis

**FIGURE 2:**
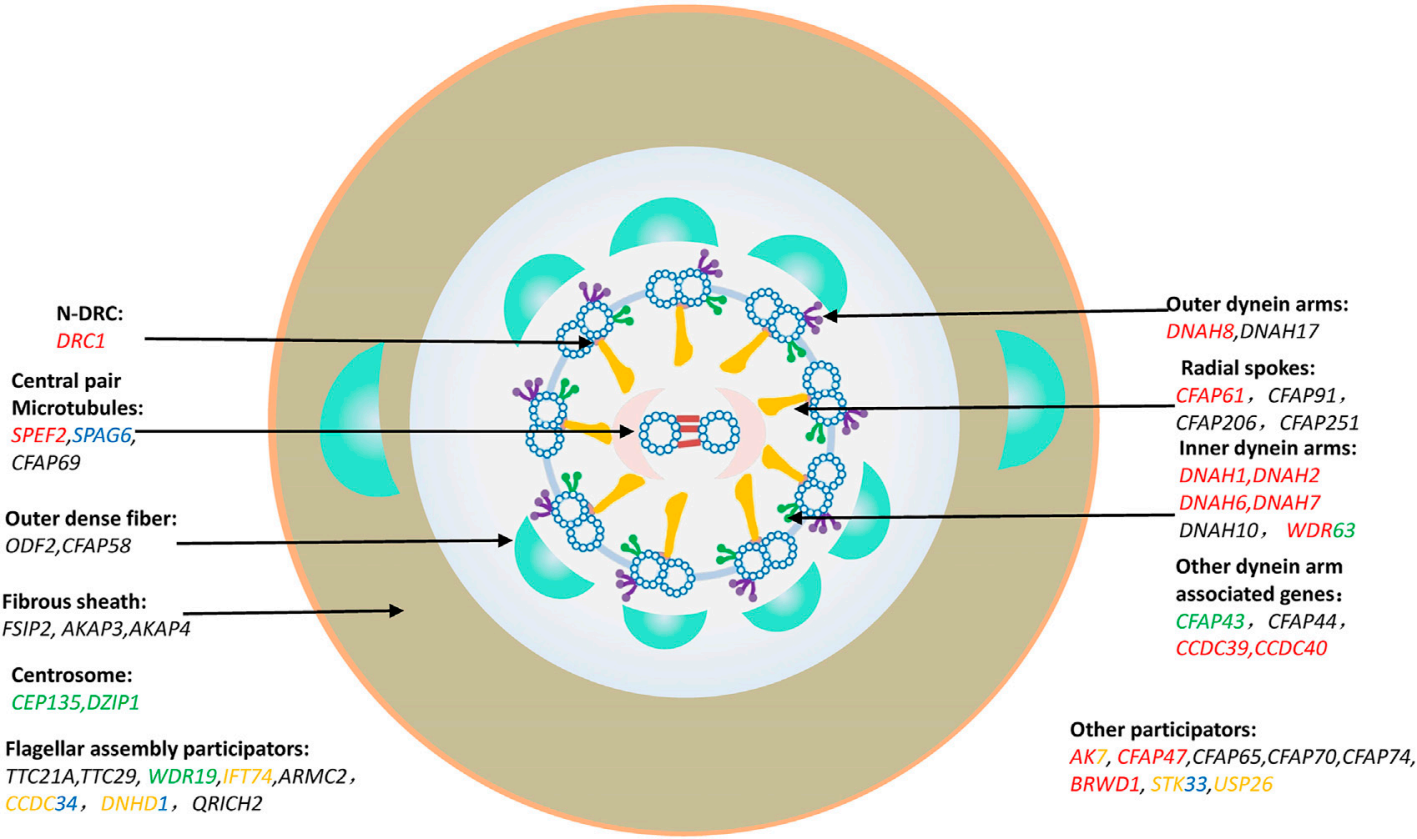
MMAF causation genes. Classification and description of MMAF pathogenic genes by protein-affected structure and mechanisms. Genes marked in red were involved in PCD; Genes marked in green were involved in neurological disorders; Genes marked in blue were involved in tumorigenesis; Genes marked in yellow were involved in rare genetic conditions, such as spondylocostal dysostosis, Joubert Syndrome, Bardet-Biedl Syndrome, Tonne-Kalscheuer syndrome, *etc.* Genes marked in multiple colors indicated that the mutations in them might cause multiple diseases, which were presented in different colors described above, respectively.

The central dogma of genetics suggested by Francis Crick in the last century (Crick, 1970) can help us understand the pathogenesis of MMAF related pathogenic genes. Thus, it is easy to understand the different sperm phenotypes caused by the different variants due to the various protein functions in spermatogenesis, including mitotic cell division, meiosis and spermiogenesis. The pathogenic genes of MMAF should be categorized according to their function and related protein domains. So far, about 43 MMAF-related genes have been reported, which account for approximately 30%–60% of MMAF patients (Touré et al., 2021). The reported MMAF causative genes are annotated in Figure 2, and the detailed pathogenic mutations are listed in Supplementary Table S1. Among those, there are 24 genes, in which pathogenic mutations may cause other diseases, such as PCD, neurological disorders, and other related ciliopathies (Figure 2).

### Flagella structure component-related genes

When introducing the molecular pathogenic mechanism of MMAF, flagella assembly can be described as being similar to building a wall with the flagella structure components acting as the “bricks,” each of which has its own unique function.

#### Central pair (CP) microtubules

Similar to steel bars in a building, microtubules are the skeletons of flagella, in which the CP is the pillar. SPEF2 is a component of the central pair complex (CPC), which has been confirmed by flagella lacking CP microtubules in *SPEF2-*deficient patients (Cindrić et al., 2020). *SPEF2* was initially found to be related to PCD (Sironen et al., 2011), and several studies have then revealed its relationship with MMAF (Liu et al., 2019b; Sha et al., 2019; Liu et al., 2020a). As a central pair complex protein, SPEF2 has been reported to be localized in the C1b projection (Zhang and Mitchell, 2004) and to interact with other CPC proteins, such as CFAP221 and CFAP54 (McKenzie and Lee, 2020). Various studies have reported that *SPAG6* is relevant in cancer (Zheng et al., 2019), and it has been recently identified as a MMAF pathogenic gene (Xu Y. et al., 2022). The mammalian *SPAG6* is the orthologue of *Chlamydomonas reinhardtii PF16,* and *PF16* gene mutations cause instability in the C1 microtubule of the CPC (Smith and Lefebvre, 1996). SPEF2 and SPAG6 are both components of the CPC, and they are widely used as microtubule markers. *CFAP69* is another pathogenic gene that encodes a CPC component, and it has been reported to be related to MMAF in two large cohorts (Dong et al., 2018; He et al., 2019). Although detailed functional reports of CFAP69 are lacking, CFAP69 has been identified in C1b/C1f subunits with SPEF2A in *Tetrahymena thermophila,* and the knockout of either *SPEF2A* or *CFAP69* results in a loss of the entire C1b projection (Joachimiak et al., 2021).

It was worth noting that these three genes are more than just structural proteins as they have been recently reported to be involved in flagellar assembly, such as intra flagellar transport (IFT) or intra manchette transport (IMT). SPEF2 has been previously reported to co-localize and interact with IFT20, which localizes in the Golgi complex of late spermatocytes and round spermatids as well as in the manchette and basal body of elongating spermatids (Sironen et al., 2010). Moreover, SPEF2 has recently been reported to co-localize with dynein 1 in the manchette and potentially act as a linker protein for dynein 1-mediated cargo transport along microtubules (Lehti et al., 2017). These observations imply that SPEF2 is involved in IMT. SPAG6 and CFAP69 both contain ARM-repeat domains, and they interact and co-localize with SPEF2, indicating that these two proteins may involve interaction with other proteins and participate in IMT (Dong et al., 2018; Liu et al., 2019c).

#### Dynein arms

Dynein arms have multiple subunit ATPases and the most important motor to drive a rhythmic ciliary beating, dividing into inner (IDAs) and outer dynein arms (ODAs). The dynein arms bind and release microtubules *via* a compact microtubule-binding domain (MTBD). Recently, using cryogenic electron microscopy (Cryo-EM), coordination of multiple ODAs has been observed to generate mechanical forces to bend microtubule doublets (Rao et al., 2021). Unlike ODAs, which mainly provide force and regulate flagellar beating, IDA mainly controls the size and shape of the bend (Yamamoto et al., 2021). Any genetic factor responsible for a defect in the dynein arm may be associated with sperm flagella anomalies, of which the most common is the dynein gene family. The defects in *DNAH1*, *DNAH5*, *DNAH6*, *DNAH7*, *DNAH8*, *DNAH9* and *DNAH11* have been demonstrated to be responsible for PCD (Watson et al., 2014; Lucas et al., 2020). *DNAH1* was the first pathogenic gene identified in MMAF patients (Ben Khelifa et al., 2014) and may be the most common MMAF pathogenic gene detected in the clinic. Coutton et al. (2018) reported that *DNAH1*mutations account for 6% of the 178 MMAF cases in their cohort. In the past 3 years, another IDA-coding gene, *DNAH2*, has been reported to be related to MMAF in three cohorts (Li Y. et al., 2019; Gao et al., 2021; Hwang et al., 2021). Interestingly, unlike *DNAH1*, there is no report for PCD patients harboring *DNAH2* variants to date. Although we classified *DNAH6* as an IDA component, immunofluorescence experiments have shown that it is localized to the head-neck junction and that its deficiency results in sperm head anomaly (Li W. et al., 2019). *DNAH7* mutations have been identified in PCD patients long ago (Zhang et al., 2002) but only recently have been found in MMAF patients (Gao et al., 2022). In addition to the sperm phenotype in PCD cases that mainly demonstrates an absence of IDAs, the MS in the sperm midpiece is detached and dispersed outside the axoneme in MMAF cases (Gao et al., 2022). The detailed structure and MTBD of DNAH7 has been observed by Cryo-EM and found to induce large distortions in the microtubule cross-sectional curvature (Lacey et al., 2019). *DNAH10* is another novel MMAF causative gene encoding an IDA component, and patients harboring *DNAH10* mutations show remarkably reduced expression levels of IDA markers, namely, DNAH1, DNAH2 and DNAH6, in their sperm (Tu et al., 2021; Li K. et al., 2022).

*DNAH8* has been identified in three MMAF cohorts, and it encodes a γ-type heavy-chain protein, which is an ODA component (Liu et al., 2020b; Yang et al., 2020; Weng et al., 2021). Another ODA component, β-type heavy chain, which is encoded by the *DNAH17* gene, acts as a MMAF causative gene (Whitfield et al., 2019; Sha et al., 2020a; Song et al., 2020; Zhang et al., 2020; Zhang G. et al., 2021; Zhang B. et al., 2021; Zheng et al., 2021; Liu G. et al., 2022). Moreover, DNAH8 or DNAH17 is absent when the other is deficient, and they are co-localized along the axoneme, indicating interaction between these two proteins (Whitfield et al., 2019; Liu et al., 2020a).

IDA is composed of heavy chains, light chains and intermediate chains, and WDR63 is the orthologue of IC140, a subunit of IDA intermediate chains, which is vital for IDA assembly in *Chlamydomonas* (Viswanadha et al., 2017). *WDR63* deficiency causes abnormal IDA assembly, resulting in MMAF (Lu et al., 2021). Co-immunoprecipitation and immunofluorescence analyses have revealed the co-expression and co-localization of WDR63 and another subunit of IDA intermediate chains, namely, WDR78. WDR63 binds WDR78 to form an IDA complex to participate in flagella assembly (Lu et al., 2021). Unfortunately, there is no case report of *WDR78* deficiency leading to abnormal sperm flagella.

After the relationship between *DNAH1* and MMAF was revealed, we reported two novel causative genes, namely *CFAP43* and *CFAP44* (Tang et al., 2017)*.* However, due to the lack of suitable antibodies, the functions of the proteins encoded by these genes have not been comprehensively studied until recently. Several phenotype studies have investigated the role of these three genes in spermatogenesis. Our group found abnormal manchette and disorganized ectoplasmic specialization at elongated spermatids in *Cfap43* knockout mice, suggesting that Cfap43 may be involved in IMT in mice (Yu et al., 2021). Functional research of these three proteins has been conducted in *Tetrahymena thermophila*. The orthologous Fap43 and Fap44 complex has been demonstrated to be located in close proximity to IDAI1 (Urbanska et al., 2018)*.* Fu et al. (2018) identified the tether and tether head (T/TH) complex in *Chlamydomonas*, which plays a critical role in normal ciliary motility, and they reported that this complex is composed of Fap43 and Fap44.

*CCDC39* and *CCDC40* have been identified in PCD patients (Becker-Heck et al., 2011; Merveille et al., 2011), and their encoded proteins form a molecular ruler to ensure establishment of 96-nm repeats, which are essential for anchoring the dynein regulatory complex (DRC) to connect the outer doublets and IDA proteins (Oda et al., 2014). Several male PCD patients harboring *CCDC39* or *CCDC40* mutations present infertility, and their sperm show the MMAF phenotype (Chen et al., 2021; Xu C. et al., 2022; Shi et al., 2022), implying the relationship between PCD and MMAF.

#### Radial spokes

In contrast to the central pair complex and dynein arms, the RS is relatively simple. The RS is composed of triplet RSs with RS3 differing from RS1 and RS2. RS1 is adjacent to the base of the inner dynein arm, while RS2 and RS3are respectively attached to the front and back of the nexin–dynein regulatory complex (N-DRC) and the calmodulin- and spoke-associated complex (CSC) (Zhu et al., 2017). Defects of several genes that encode CSC components have been reported to be related to MMAF, including *CFAP61* (Ma A. et al., 2021; Liu M. et al., 2021; Hu et al., 2022), *CFAP91* (Martinez et al., 2020), *CFAP206* (Shen et al., 2021) and *CFAP251* (Auguste et al., 2018; Kherraf et al., 2018; Li W. et al., 2019; Wang J. et al., 2021). Early in 2007, Dymek and Smith conducted immunoprecipitation experiments using anti-CaM antibodies and found that the CSC is comprised of three polypeptides, namely, CaM-IP2, CaM-IP3 and CaM-IP4, which are also termed as CFAP61, CFAP91 and CFAP251, respectively (Dymek and Smith, 2007). In human cases, the defects in these three genes cause a similar sperm ultrastructure phenotype, including absence of CP, disorganized axonemal components and periaxonemal structural defects. Ma et al. observed a complete absence of CFAP251in the flagella of patients harboring *CFAP61* mutations, suggesting that CFAP61 may play a more predominant role in CSC. It is worth noting that in patients with *CFAP61* and *CFAP91* mutations, the flagellum CP shows a similar incorrect rotation or loss, indicating the key role of CSC in correct orientation and stability of the CP (Martinez et al., 2020; Ma A. et al., 2021). In addition to the common dysmorphic features, severe MS abnormalities have been reported in patients with *CFAP251* mutations, which are attributed to the potential effects of CFAP251 on the extension of the MS along the midpiece of the spermatozoon flagellum (Auguste et al., 2018). *CFAP206* encodes a microtubule-docking adapter for RS and IDA rather than a CSC component, and it has recently been found be related to MMAF (Shen et al., 2021). Immunofluorescence analysis has indicated that CFAP251 appears completely absent in the sperm cells of patients harboring *CFAP206* mutations, indicating the important role of CFAP206 in CSC assembly or stability (Shen et al., 2021). Beckers et al. (2020) generated polyclonal antibodies to study CFAP206 localization and reported that it is located on the axoneme and basal bodies. Interruption of *CFAP206* does not have a significant effect on sperm flagella morphology in mutant mice, but it significantly reduces the sperm motility by affecting the flagellar beat frequency. Knockout of the orthologous *FAP206* in the ciliate *Tetrahymena* causes the loss of RS2 and dynein c with no or mild effect on RS1 and RS3, suggesting that FAP206 is part of the front prong and attaches RS2 and dynein c to the microtubule (Vasudevan et al., 2015).

#### Nexin-dynein regulatory complex

The N-DRC is an axonemal structure critical for regulating dynein motors, and it is divided into two regions, namely the linker and base plate (Wirschell et al., 2013). Although mutations of *DRC1*, also called *CCDC164*, have mainly been associated with PCD, they have recently been identified in MMAF patients. DRC1 is a highly conserved structural component of the N-DRC, an axonemal structure critical for regulation of dynein motors (Wirschell et al., 2013). Using cryo-electron tomography and structural labeling, the precise localization of DRC1 has been observed, starting from the linker domain, spanning across the base plate and ending near the hole on the inner junction, suggesting that DRC1is the backbone of the N-DRC (Oda et al., 2015). *DRC1*deficiency results in axoneme structure abnormalities. Moreover, Zhang et al. attempted to generate *Drc1* knockout mice on a C57BL/6 background for animal model validation but failed due to hydrocephaly and postnatal death.

#### Periaxonemal structures

On the one hand, periaxonemal structures (ODFs and the FS in the midpiece as well as ODFs and the MS in the principal piece) provide additional flagellar stiffness and create the functional effect of increasing bend wavelength; on the other hand, they maintain the integrity of the central axoneme (Lindemann and Lesich, 2016). ODF2 is one of the major proteins in ODFs in sperm, and in somatic cells, it is a component of the centrosome located in the appendages of the mother centrioles (Schweizer and Hoyer-Fender, 2009). The study by Zhu et al. (2022) was the first and only report to date on a MMAF patient with an *ODF2* mutation. Due to the function of ODF2 in the basal bodies in ciliated tissues, homozygous *Odf2* knockout mice experience early postnatal death due to PCD or impaired gastrointestinal motility, but heterozygous *Odf2* ^+/−^ mice are infertile due to haplo insufficiency with decapitated and decaudated spermatozoa (Ito et al., 2019), which is similar to the report in humans by Zhu et al. *CFAP58* has been identified in two Chinese cohorts of MMAF patients (He et al., 2020; Sha et al., 2021); more than 90% of the axonemal cross-sections of patients harboring *CFAP58* mutations are abnormal, and the number of ODFs is almost doubled in some of the flagella, indicating the function of CFAP58 in both axoneme and periaxonemal structures. CFAP58 has been reported to co-localize and interact with ODF2, playing an important role in sperm midpiece assembly by regulating the Notch signaling pathway (Li W. et al., 2020).

Abnormal FSs were observed in most MMAF cases. However, among the reported causative genes, few are directly related to FSs. *FSIP2*, which encodes a FS-interacting protein, is involved in FS assembly, and *FSIP2* defects have been reported to be related to MMAF (Martinez et al., 2018; Liu Y. et al., 2019; Liu S. et al., 2021; Fang et al., 2021; Hou et al., 2022; Yuan et al., 2022; Zheng et al., 2022). In patients harboring *FSIP2* mutations, the defects in sperm are not limited to abnormalities in the FS but also cause abnormalities in the microtubule skeleton. A recent study has reported that *FSIP2* mutant sperm show a globozoospermia phenotype in addition to flagella defects, and FSIP2 localizes in the acrosome and interacts with proteins associated with acrosome formation, indicating the potential function of FSIP2 in acrosome development (Zheng et al., 2022), which is consistent with single-cell RNA-seq data (Fang et al., 2021). FSIP2 has been demonstrated to interact with two other FS proteins, namely, AKAP3 and AKAP4, which are encoded by MMAF causative genes (Zhang G. et al., 2021; Liu Z. et al., 2022). In 2005, the possible relationship between DFS and partial deletions in the *AKAP3* and *AKAP4* genes was revealed (Baccetti et al., 2005b). AKAP3 and AKAP4 are the most abundant proteins in the FS, and they are involved in the protein kinase A (PKA) signaling pathway and participate in the regulation of various cellular structure subunits (Burton and McKnight, 2007). The proteins that interact with AKAP3 and AKAP4 are widely distributed among various sperm cytoplasm subunits, including the nucleus, acrosome, endoplasmic reticulum/Golgi and plasma membrane. Due to the multiple mechanisms of these two proteins in spermatogenesis, the defects caused by these genes vary, including FS and CP abnormalities as well as a slightly higher rate of abnormal acrosomal morphology. In addition, Xu et al. (2020) reported that*AKAP3* and *AKAP4* mutations in mice not only change the integrity of sperm structure by causing mislocalization of sperm proteins but also lead to global changes of sperm proteomes.

#### Centrosome

It is well-known that the centrosome is a major organizer of the cytoskeleton in animal cells, ensuring precise organization of the mitotic apparatus and chromosome segregation, and the centrosome has additional functions in flagella assembly. The centrosome is composed of a pair of centrioles with structural and functional asymmetry, called mother and daughter centrioles. In sperm flagella, the mother centriole turns into a basal body, which is involved in growing the axoneme and controlling the entry of flagellar complexes (Bornens, 2012). *CEP135*, encoding a centrosomal protein, plays a role in centriole biogenesis and CP assembly, and it has been identified in a MMAF patient (Sha Y. W. et al., 2017). In *Chlamydomonas* and *Paramecium*, BLD10, the CEP135 orthologue, localizes close to the CP at the spoke tips of the cartwheel, while in humans and *Drosophila*, it localizes in centrioles but not in the axoneme (Carvalho-Santos et al., 2012). Unfortunately, the study reported by Sha et al. did not include sperm flagella ultrastructure data, preventing the ability to determine the impact of *CEP135*mutation on human axoneme assembly. *DZIP1*encodes a zinc finger- and coiled-coil-containing protein, representing another centrosome protein identified in MMAF patients (Lv et al., 2020). Immunofluorescence staining of DZIP1 in humans has indicated the localization of DZIP1 in the sperm head and neck, and immunofluorescence staining of the centriolar marker, Centrin1, has indicated that mutant spermatozoa show abnormalities, including two centriolar dots with abnormal angle, no concentrated dot or more than two centriolar dots, compared to the control individual. Using cultured human cells, DZIP1 has been found to interact with BBSome to form the BBSome-Dzip1-PCM1 complex, which regulates the ciliary translocation of the BBSome and ultimately cilia/flagella assembly (Zhang et al., 2017).

### Genes encoding proteins that interact with other proteins to participate in flagellar assembly

There are many proteins involved in IFT and IMT, which are two important processes in flagellar protein transportation. IFT is a highly conserved bidirectional cargo delivery system, relying on motor proteins within IFT complexes, which are divided into two subcomplexes, namely, IFT-A and IFT-B (Hao and Scholey, 2009). Defects in the proteins belonging to or interacting with the IFT complexes are harmful to the assembly of flagella and may cause MMAF. IMT is another protein transport system that involves a transient microtubule and actin-based structure called manchette, which is present in step 2 spermatids in humans. IMT participates not only in flagella assembly but also nuclear remodeling, and proteins involved in IMT may overlap with those involved in IFT (Lehti and Sironen, 2016). Therefore, abnormalities in IMT-related proteins may also lead to MMAF.

*TTC21A*, also termed as *IFT139A*, encodes an IFT complex component containing tetratricopeptide repeat (TPR) domains, and it is a MMAF causative gene (Liu C. et al., 2019; Liu C. et al., 2022). *IFT139* encodes only one protein in *Chlamydomonas* but encodes two orthologues, namely, TTC21A and TTC21B, in mice and humans, which interact with each other and play a critical role in many procedures, including regulating retrograde IFT, ciliogenesis and cilia disassembly (Wang W. L. et al., 2020). *TTC29*, another TPR-containing protein, has been identified as a MMAF causative gene in two large cohorts (Liu et al., 2019a; Lorès et al., 2019). Unlike TTC21A, TTC29 has been suggested to be a potential member of the IFT-B complex and participate in anterograde IFT (Chung et al., 2014; Beneke et al., 2019). *WDR19*, also termed as *IFT144*, encodes a protein acting as a core component in IFT-A (Efimenko et al., 2006), and it has been reported to be related to MMAF (Ni et al., 2020). In cranio ectodermal dysplasia, *WDR19* defects have been found to be harmful to interactions with other IFT-A subunits and the IFT-B complex, thereby affecting cilia/flagella assembly (Ishida et al., 2021). *IFT74*, another IFT core component-encoding gene, has been linked to Bardet-Biedl syndrome, but Lorès et al. (2021) reported that it is a causative gene of MMAF without clinical signs of Bardet-Biedl syndrome. Prior to these studies, IFT74 has been indicated to be essential for spermatogenesis in mice as demonstrated with *Ift74* knockout mice (Shi et al., 2019)*.* By studying *Chlamydomonas* mutants, the IFT81 and IFT74 N-termini have been demonstrated to form the main module for IFT of tubulin, indicating their crucial role in flagellar assembly (Kubo et al., 2016). Mutations in *ARMC2* have been identified in MMAF patients in several cases (Coutton et al., 2019; Khan et al., 2021; Wang Y. et al., 2022), and the function of the encoded protein has been recently reported. In *Chlamydomonas*, the orthologue of ARMC2, PF27, co-migrates with the RSP3 spoke protein on anterograde trains, indicating that AMRC2 is not an IFT complex component but an adapter required for IFT of RSs to ensure their assembly along flagella (Lechtreck et al., 2022).

Other proteins have been identified that may not be obvious components of the IFT complex or an adapter but have been suggested to play an important role in flagella assembly. CCDC34, containing a coiled-coil domain, has been found to be defective in some MMAF patients (Cong et al., 2022). Immunofluorescence staining has demonstrated that the IFT-B related proteins, IFT20 and IFT52, are almost absent in the spermatozoa in a patient harboring a *CCDC34* mutation, whereas the IFT-A related IFT140 protein shows no obvious change, indicating that CCDC34 may influence sperm flagellar formation through anterograde IFT. Another coiled-coil domain containing protein, CCDC35, also called DNHD1, has been found to be defective in MMAF patients (Tan et al., 2022). There is little research on the role of DNHD1 in spermatogenesis, but the potential role of DNHD1 on IFT has been speculated based on the *DNHD1* mutant sperm phenotypes, including the absence and abnormalities of CP and MS. Some genes had no clear evidence to prove that they participated in IFT, but their key role in flagellum assembly could be understood through protein interaction prediction. Mutations in *QRICH2* have been identified in MMAF infertile men (Kherraf et al., 2019; Shen et al., 2019). In a functional study, Shen et al. reported that QRICH2 inhibits the ubiquitination degradation of AKAP3 and ROPN1 as well as increases CABYR mRNA levels through a TSSK4/ODF2-mediated pathway to participate in the formation and stabilization of flagellar ultrastructure.

### Genes that encode protein with unclear or undefined function

In this section, we will briefly introduce some causative genes but will not classify them in this review because their functions are still debatable.

*AK7*, encoding an adenylate kinase, is a rare enzyme-encoded gene in the MMAF causative gene family (Lorès et al., 2018; Xiang et al., 2022). As an enzyme, AK7 catalyzes the reversible transphosphorylation reaction of two molecules of ADP to one molecule each of ATP and AMP. AK7 has been identified in mouse sperm and suggested to be related to PCD (Fernandez-Gonzalez et al., 2009). In humans, AK7 is localized to the sperm flagellum, but its precise localization and function in sperm still remain to be determined.

To date, only one report on the relationship between mutations of *CFAP47* and MMAF has been published (Liu et al., 2021d). The detailed molecular mechanism of CFAP47 in spermatogenesis is unclear, but Liu et al. (2021d) suggested an interaction between CFAP47 and CFAP65 based on immunostaining and a co-immunoprecipitation assay. *CFAP65* has been characterized as a causative gene in MMAF patients, and it has been identified together with *CFAP43* and *CFAP44* (Tang et al., 2017) followed by several case reports (Wang et al., 2019; Zhang et al., 2019; Li Z. Z. et al., 2020). Previous studies on Fap57, the orthologue of human CFAP65, cellular location in *Tetrahymena thermophila* have demonstrated that it interacts with Fap43 and Fap44, indicating a potential location and function of CFAP65 in IDA (Urbanska et al., 2018). However, this interaction has not been observed in mammals, and recent research has suggested a different localization of this protein in *Chlamydomonas* where Fap65 and Fap147 co-localize and co-immunoprecipitate with FAP70, indicating that these three proteins are components of the C2a projection. In humans, immunofluorescence staining of CFAP65 has been visualized in the acrosome and midpiece of spermatozoa, indicating its potential function in acrosome biogenesis and flagellar assembly, which has been demonstrated in subsequent animal experiments (Wang et al., 2019). A mouse model has indicated that Cfap65 plays an important role in sperm head shaping, acrosome biogenesis and MS assembly (Wang W. et al., 2021), while a previous study in *Xenopus laevis* embryos, Cfap65 has been indicated to cooperate with IFT-B complex components and regulate multiciliogenesis *via* an IFT mechanism (Zhao et al., 2022). *CFAP70* is another MMAF-related gene that encodes a protein with unclear functions (Beurois et al., 2019). CFAP70 contains a cluster of tetratricopeptide repeat (TPR) domains, similar to many components involved in IFT. Using a mouse model and *Chlamydomonas*, Noritoshi et al. found that CFAP70 is a novel regulatory component of the ODA and that the N-terminus is important for its localization (Shamoto et al., 2018). In MMAF patients harboring *CFAP70* mutations, however, the mutations not only affect the DNAI2 ODA marker but also the SPAG6 CPC marker, which is absent, indicating the impact of CFAP70 on both ultrastructural units. A recent study has suggested that *Chlamydomonas* FAP70 is a component of the C2a ciliary central apparatus projection, which is consistent with the finding in humans (Hou et al., 2021). Similar to *CFAP47*, only one MMAF case report exists for *CFAP74* variants (Sha et al., 2020b). Although *CFAP74* is highly expressed in the testis and down regulated in maturation arrest and oligospermia cases (Razavi et al., 2017), the detailed mechanism of CFAP47 in spermatogenesis and localization in sperm remain unknown.

*BRWD1* has been identified in patients with both MMAF and PCD phenotypes. In the sperm of patients harboring *BRWD1* mutations, IDAs and ODAs are absent, while the outer doublet microtubule number and the CPC are normal, suggesting a potential function of BRWD1 in dynein arm assembly but without clear evidence (Guo et al., 2021). The sperm of *Brwd1*mutant mice exhibit multiple morphological defects, suggesting that *BRWD1*is essential for haploid gene expression during post meiotic germ cell differentiation during spermiogenesis (Pattabiraman et al., 2015).

*STK33,* encoding a serine/threonine kinase, has been reported to be related to MMAF in one study (Ma H. et al., 2021), but the detailed function of STK33 is unclear. Knockout mice have indicated that STK33 is critical for manchette formation, and STK33 knockout leads to abnormal head shaping and severe impairment of tail development in sperm (Martins et al., 2018). Nevertheless, the specific molecular pathway and mechanism of STK33 acting on sperm flagellogenesis remain unclear.

*USP26* is a single-exon gene located on the X chromosome that encodes a deubiquitylating enzyme belonging to the ubiquitin specific protease family (Wang et al., 2001). The relationship of USP26 with male fertility has been investigated by mainly focusing on the mutations resulting in azoospermia (Xia et al., 2014; Ma et al., 2016; Arafat et al., 2020). However, an opposite conclusion has suggested that *USP26* is not related to male fertility (Zhang et al., 2015) and is not essential for mouse gametogenesis (Felipe-Medina et al., 2019). Using mice from different genetic backgrounds, Sakai et al. (2019) attributed this different impact of USP26 on spermatogenesis to the genetic background. However, the mechanism of USP26 causing MMAF remains unknown.

### Inheritance pattern

As shown in Supplementary Table S1, MMAF is an autosomal recessive genetic disease in most cases. However, further research has revealed additional pathogenic genes and their new inheritance patterns. In the report of *ODF2*, MMAF is caused by a single heterozygous mutation, which has been proven by the mice model (Zhu et al., 2022), suggesting that this phenomenon is attributed to dose-dependent effects, i.e., products derived from different alleles of heterozygotes expressed after allele separation would be distributed at a concentration gradient in these cells, leading to varying phenotypes. Apart from the rare pattern, additional X-linked genes have been classified in the MMAF-related gene family, including *AKAP4* (Zhang B. et al., 2021), *CFAP47* (Liu S. et al., 2021) and *USP26* (Liu et al., 2021d), which has increased the difficulty of clinical genetic counseling. In most instances, the offspring of the MMAF patient is only a mutation carrier and would not harm the next generation if the future partner is normal. However, if the mutant gene is an X-linked gene, the first-generation offspring of the MMAF patient may be carriers or have the disease depending on their gender, female or male, respectively. Moreover, female carriers can pass the mutation onto their male children causing MMAF. Whether pre-implantation genetic testing (PGT) should be used to block offspring carrying genetic risk in this circumstance is worth considering and debating. In a recent study, we reported a MMAF case caused by a splicing mutation in *CFAP251*, in which the patient’s father was normal and mother was a heterozygous carrier. The mode of inheritance has been demonstrated to be UPD, an inheritance mode of two homologous chromosomes from the same parent (Wang J. et al., 2021). The mechanism of UPD involves several circumstances, including isodisomy from independent mitotic errors, segmental loss of heterozygosity and micronuclei (Benn, 2021). UPD also challenges clinical counsel and treatment. In common cases, if one parent is normal, even if the other parent is a carrier, the offspring would be predicted to not be at risk of MMAF. However, similar to the case of *ODF2*, UPD may cause a heterozygous mutation in one parent, requiring adequate risk estimates and explanations in clinical consultations.

### Overlap of MMAF with other ciliary disorders

Due to the structural similarity between sperm flagella and cilia in other organs, such as respiratory tract and brain ventricle, the genes responsible for MMAF partially overlap with those of chronic respiratory symptoms, such as PCD, and some brain diseases (Nsota Mbango et al., 2019; Wang W. et al., 2020). Tu et al. (2020) demonstrated the involvement of *SPEF2* in both sperm flagella and somatic cilia in humans, and they speculated that MMAF may be a phenotypic variation of the classical forms of PCD. Morimoto et al. (2019) identified a nonsense mutation in *CFAP43* in multiple individuals with normal-pressure hydrocephalus and observed that CFAP43 has impacts not only on the testis but also other tissues, including the ventricles of the brain and trachea. In addition, several MMAF causative genes have been reported to be related to tumorigenesis (Kong et al., 2017; Zhu et al., 2019; Li N. et al., 2022). Over the years, different phenotypes have been shown to be caused by different mutations in the same gene, indicating the need for a review, in which we believe that MMAF, PCD and other ciliary diseases are completely homologous diseases with different phenotypes. The results depend entirely on the different effects of different mutations on protein function and the subtle differences in cilia/flagellar structural components and assembly processes in different tissues. Consistent with our thoughts, a previous study on ODF2 and CFAP58 has demonstrated that some proteins, such as ODF2, perform distinct functions in different organelles (Li W. et al., 2020). However, more studies are required before this phenomenon will be fully explained.

## ICSI outcome of MMAF patients

As previously mentioned, ICSI may be the only way to help MMAF patients obtain their offspring. Moreover, to date, there is no report of spontaneous reproduction of MMAF patients due to awkward sperm motility and morphology. In 1997, Olmedo et al. (1997) reported a successful ICSI performed with immotile spermatozoa from a MMAF patient. Recently, Hu et al. (2021) reported that a patient harboring a *DNAH1* mutation utilized IVF to successfully obtain a healthy baby, but the detailed data is lacking, thereby making the result less convincing. The reported ICSI outcomes of MMAF patients are listed in Supplementary Table S2. Most variants do not harm embryo development or cause failure in pregnancy. However, failure of ICSI in MMAF patients is often related to several genes. *CEP135* is the most cited gene when discussing failed ICSI outcome because it encodes a protein that is critical to the formation of centrioles, which play an important role in chromosomal stability, cell division and embryonic development (González-Martínez et al., 2021). To date, there is no report on a successful ICSI outcome for patients harboring *CFAP65* mutations. A recent study has reported that CFAP65 localizes to centrioles in the cilium in *Xenopus* and plays a key role in basal body migration during multiciliogenesis (Zhao et al., 2022). *CFAP65* deficiency in mice results in abnormalities in sperm head shaping and acrosome biogenesis, which is crucial to subsequent embryonic development after sperm injection (Wang W. et al., 2021). Before the report by Song et al. (2020), only one individual harboring *DNAH17* mutations has achieved successful ICSI (Whitfield et al., 2019; Zheng et al., 2021). The patient in the reports by Song et al. (2020) underwent ICSI followed by an artificial oocyte activation (AOA) cycle, which resulted in a successful pregnancy. In another case, the patient’s spouse’s oocytes were unable to be fertilized even with ICSI-AOA (Jia et al., 2021). Based on the protein function of DNAH17 on the ODA in the axoneme, it is difficult to associate DNAH17 with embryo development. Therefore, DNAH17 may play a potential role in other sperm ultrastructure components to affect the outcome of ICSI. Other studies have reported sporadic ICSI failure outcomes, but they do not account for the majority of cases. Because fertilization and embryo development are intricate processes affected by multiple factors, it is difficult to determine whether the failure is caused by pathogenic mutations or other factors. At present, MMAF patients in our center all had good ICSI outcomes, unless the female spouse had reproductive diseases, which might be due to the fact that most of these patients carried *DNAH1* and *CFAP43* mutations. Recently, the wife of a patient in our center with *CFAP65* mutation has successfully achieved clinical pregnancy, but has not yet delivered, which is an important case that we would pay more attention to in the future. Because most MMAF patients have good ICSI outcomes, many doctors without relevant experience will skip genetic diagnosis and directly conduct ICSI, leading to the potential failure, which should be avoided. We believe that accurate treatment based on different pathogenic genes and mutations is the future development direction of assisted reproductive therapy for MMAF patients.

## Conclusion

With the development of detection technology and genetic studies, MMAF has attracted increasing attention from clinical doctors. At the conclusion of this literature review, new case reports continued to emerge. Nowadays, the standardization of clinical diagnosis and treatment is imperative. To better understand the pathogenic mechanism of MMAF, more basic functional studies as well as new case reports are needed. With better understanding of MMAF, the name of such disorders may be replaced by more precise terms. In the future, the diagnosis and treatment of MMAF should be determined by the specific phenotype and genotype of each individual patient, and this literature review will hopefully provide reference value for clinical practice.
