# Supplementary material for: Clinical detection, diagnosis and treatment of morphological abnormalities of sperm flagella: A review of literature
Source: Front Genet. 2022 Nov 8;13:1034951. doi: 10.3389/fgene.2022.1034951 (PMC9679630; doi:10.3389/fgene.2022.1034951)
Supplement: Supplementary file 1 [file DataSheet1.docx]

Supplementary Material

**
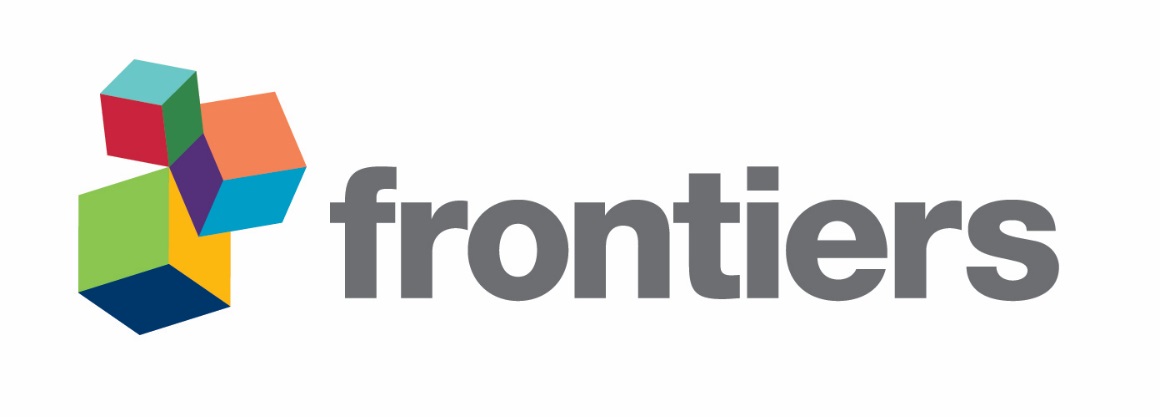
**

**Supplementary Table1. Mutations identified in infertile MMAF patients and reported in the literature**

| **Protein Function** | | **Gene** | **Major phenotypic features** | **cDNA Variation** | **Amino acid variation** | **Variant type** | **Origin** | **Allelic status** | **Exac Mutation frequency** | **References PMID** |
| --- | --- | --- | --- | --- | --- | --- | --- | --- | --- | --- |
| Flagella structure component related genes | Central pair Microtubules | *SPEF2* | No spermatozoa with progressive motility  Absence of CP(9+0) (main defect) | c.910C>T | p.Arg304* | Nonsense | China | Homozygous | 8.2×10^−6^ | 31048344 |
|  |  |  |  | c.3400delA | p.Ile1134Serfs*13 | Frameshift |  | Homozygous | 8.3×10^−6^ |  |
|  |  |  |  | c.3240delT | p.Phe1080Leufs*2 | Frameshift | Iran | Homozygous | NA |  |
|  |  |  | No spermatozoa with progressive motility  Absent , short and coiled flagella were the most frequently observed  Absence of CP(9+0) (main defect)  Disorganized peripheral microtubule doublets  Misarranged MS | c.2734delC | p.Pro912fs | Frameshift | China | Heterozygous | NA | 31278745 |
|  |  |  |  | c.4952delT | p.Val1651fs | Frameshift |  | Heterozygous | NA |  |
|  |  |  | No spermatozoa with progressive motility  Absent and short flagella were the most frequently observed  Absence of CP(9+0) (main defect)  Peri axonemal structures were disarranged  Fibrous sheaths are fragmented or disorganized | c.12delC | p.Ile4fs | Frameshift | China | Heterozygous | NA | 31151990 |
|  |  |  |  | c.1745-2A>G |  | Splicing variant |  | Heterozygous | 7.0×10^−6^ |  |
|  |  |  |  | c.4102G>T | p.Glu1368* | Nonsens |  | Heterozygous | NA |  |
|  |  |  |  | c.4323dupA | p.Ile1441fs | Frameshift |  | Heterozygous | NA |  |
|  |  | *SPAG6* | Spermatozoa with progressive motility less than 1%  Short and coiled flagella were most frequently observed  Absence of CP(9+0) (main defect)  peripheral microtubule doublets and ODFs were translocated disorganized | c.308C > A | p. Ala103Asp | Missense | China | Homozygous | NA | 35232447 |
|  |  |  |  | c. 585delA | p. Lys196Serfs*6 | Frameshift |  | Homozygous | 8.25x 10^-6^ |  |
|  |  | *CFAP69* | Spermatozoa with progressive motility less than 1%  Short and coiled flagella were most frequently observed  Absence of CP(9+0) (main defect) | c.1069_1070insAC | p.Leu357Hisfs11* | Frameshift | China | Homozygous | 8.0×10^−6^ | 30415212 |
|  |  |  |  | c.647G>A | p.Trp216* | Nonsense |  | Homozygous | 7.0×10^−6^ |  |
|  |  |  | Very low sperm concentration, total sperm counts and motility  Besides flagella defects, a high rate of head malformations, in particular thin heads and an abnormal acrosomal region | c.860+1G>A | - | Splicing variant | Iran | Homozygous | NA | 29606301 |
|  |  |  |  | c.763C>T | p.Gln255Ter | Missense | North Africa | Homozygous | NA |  |
|  | Dynein arms | *DNAH1* | Normal semen volume and normal sperm concentration with low sperm motility of 0.67%  Short and coiled flagella were most frequently observed  Absence of IDA and RS, displaced ODFs and microtubule doublets | c.1336G>C | p.Glu446Gln | Missense | China | Heterozygous | 7.0x 10^-6^ | 33989052 |
|  |  |  |  | c.2912G>A | p.Arg971His | Missense |  | Heterozygous | 0.000198 |  |
|  |  |  | Very low sperm concentration, and motility  Absence of CP(9+0) and disarranged ODFs | c. 8170C>T | p. Arg2724* | Nonsense | China | Heterozygous | NA | 33968654 |
|  |  |  |  | c. 4670C>T | p. Thr1557Met | Missense |  | Heterozygous | 0.000033 |  |
|  |  |  | Low sperm concentration  No spermatozoa with progressive motility | c.2016T>G | p.Tyr672* | Nonsense | China | Heterozygous | NA | 34487528 |
|  |  |  |  | c.6017T>G | p.Val2006Gly | Missense |  | Heterozygous | NA |  |
|  |  |  |  | c.2610G>A | p.Trp870* | Nonsense |  | Homozygous | NA |  |
|  |  |  | Low motility but relatively high viability  Absence of CP(9+0) (main defect)  Disorganized FS and ODFs | c.11726_11727delCT | p.Pro3909Argfs*33 | Frameshift | China | Homozygous | 0.000083 | 27573432 |
|  |  |  | Approximately 30% motility  Short flagella were most frequently observed | c.3860T>G | p.Val1287Gly | Missense | Italy | Homozygous | NA | 27798045 |
|  |  |  | Very low motility (<5%)  Short flagella were most frequently observed | c.8626-1G>A | - | Splicing variant variant | Italy | Homozygous | NA |  |
|  |  |  | Very low motility ( <2%)  Complete disorganization of the IDA  Absence of CP(9+0)  Disorganized FS | c.11788-1G>A | p.Gly3930Alafs*120 | Frameshift | Tunis | Homozygous | NA | 24360805 |
|  |  |  | No spermatozoa with motility | c.12796 T>C | p.4266Glnext*21 | Frameshift | Algeria | Homozygous | NA |  |
|  |  |  | 35% motility and 6% morphologically normal spermatozoa | c.3877G>A | p.Asp1293Asn | Missense | Algeria | Homozygous | 0.000149 |  |
|  |  |  | Very low motility (0.5%) | c.5094+1G>A | p.Leu1700Serfs72 | Frameshift | Algeria | Homozygous | NA |  |
|  |  |  | No spermatozoa with motility  Absence of CP(9+0) (main defect)  Disorganized FS and missing microtubule doublets | c.7646_7647InsC | p.Asn2549Glnfs*61 | Frameshift | Pakistan | Homozygous | NA | 34867808 |
|  |  |  | Very low motility ( < 1%) | c.6212T>G | p.Cys1789Tyr | Missense |  | Homozygous | 0.00014 |  |
|  |  |  | Very low motility (2.6 ± 4.2%)  Short, absent and irregular caliber flagella were most frequently observed | c.7531delC | p.Gln2511Serfs*27 | Frameshift | Tunis | Homozygous | NA | 29449551 |
|  |  |  |  | c.2127dup | p.Ile710Hisfs*4 | Frameshift | Tunis | Homozygous | NA |  |
|  |  |  |  | c.4744_4752delCCAGCTGGC | p.Pro1582_Gly1584del | In-frame deletion, unknown effect | Tunis | Homozygous | NA |  |
|  |  |  |  | c.4531G>A | p.Val1511Met | Nonsense | Tunis | Heterozygou | 0.018853 |  |
|  |  |  |  | c.4642C>G | p.Leu1548Val | Synonymous variant. 5’ cryptic splice site. | Iran | Heterozygou | NA |  |
|  |  |  |  | c.7153T>A | p.Trp2385Arg | Missense | Iran | Heterozygous | NA |  |
|  |  |  |  | c.9505C>G | p.Arg3169Gly | Missense | France | Heterozygous | 8.0x 10^-6^ |  |
|  |  |  | No spermatozoa with progressive motility  Short and absent flagella were most frequently observed | c.5105G>C | p.Arg1702Pro | Missense | China | Heterozygous | NA | 31676830 |
|  |  |  |  | c.11726_11727del | p.Pro3909fs | Frameshift |  | Heterozygous | 0.000083 |  |
|  |  |  |  | c.8151-1G>C | - | Splicing variant |  | Heterozygous | NA |  |
|  |  |  |  | c.12286C>T | p.Arg4096Cys | Missense |  | Heterozygous | 0.000025 |  |
|  |  | *DNAH2* | Very low motility (≤6%)  Short and absent flagella were most frequently observed  Defects of CP(9+0,9+1,disorder) (main defect)  Missing IDA | c.2116A>C | p. Lys706Gln | Missense | China | Heterozygous | 0.000173 | 33771466 |
|  |  |  |  | c.11635C>T | p. Arg3879Trp | Missense |  | Heterozygous | 0.000231 |  |
|  |  |  |  | c.5507A>G | p. Lys1836Arg | Missense |  | Heterozygous | 0.000115 |  |
|  |  |  |  | c.9291G>T | p. Glu3097Asp | Missense |  | Heterozygous | 0.000107 |  |
|  |  |  |  | c.4774G>A | p. Glu1592Lys | Missense |  | Heterozygous | 8.0x 10^-6^ |  |
|  |  |  |  | c.5771G>C | p. Arg1924Pro | Missense |  | Heterozygous | NA |  |
|  |  |  | Very low motility (≤7%)  Several morphologically normal sperm (8-20%)  Mild defects in sperm head | c.12720G > T | p.Trp4240Cys | Missense | Pakistan | Homozygous | NA | 33968937 |
|  |  |  | Very low progressive motility (≤1%)  Short, coiled and absent sperm flagella were most frequently observed (85%-92%)  Absence of CP(9+0)  Disorganized, redundant, missing or fragmented peripheral microtubule doublets/ODF  Disorganized, partitional or hypertrophic MS | c.9298C>T | p.Arg3100Trp | Missense | China | Homozygous | 0.000016 | 30811583 |
|  |  |  |  | c.5770C>T | p.Arg1924Cys | Missense |  | Heterozygous | 0.000016 |  |
|  |  |  |  | c.11500C>T | p.Arg3834* | Nonsense |  | Heterozygous | 8.0x 10^-6^ |  |
|  |  |  |  | c.6960C>A | p.Ser2320Arg | Missense |  | Heterozygous | 0.000033 |  |
|  |  |  |  | c.11503T>C | p.Ser3835Prp | Missense |  | Heterozygous | 0.000016 |  |
|  |  | *DNAH6* | Very low progressive motility (0-5.3%)  With several normal flagella (2.4-6.0%)  Short ,absent and coiled flagella were most frequently observed  Absence of CP(9+0) (main defect)  Disorganized FS or MS | c.6582C>A | p.Asp2194Glu | Missense | China | Heterozygous | NA | 31676830 |
|  |  |  |  | c.11258G>A | p.Gly3753Asp | Missense |  | Heterozygous | NA |  |
|  |  |  |  | c.10025G>A | p.Arg3342His | Missense |  | Heterozygous | 0.000121 |  |
|  |  |  |  | c.2823dupT | p.Ser941fs | Frameshift |  | Heterozygous | 7.0x 10^-6^ |  |
|  |  | *DNAH7* | Very low progressive motility (5.3%)  Short and coiled flagella were most frequently observed  Severe IDAs loss (main defect)  Disarranged MS | c.2478dupA | p.V827Sfs*20 | Frameshift | China | Homozygous | 0.000074 | 35543642 |
|  |  | *DNAH8* | Very low progressive motility(≤4%)  Coiled flagella were most frequently observed  With quite a few normal flagella (24.5-32.3%)  Disorganized peripheral microtubule doublets and ODFs, missing or disassembled ODAs, and absent CP | c.11771C>T | p.Thr3924Met | Missense | China | Heterozygous | 0.005098 | 32619401 |
|  |  |  |  | c.6689A>G | p.Lys2230Arg | Missense |  | Heterozygous | 7.0x 10^-6^ |  |
|  |  |  |  | c.9427C>T | p.Arg3143Cys | Missense |  | Heterozygous | 0.000066 |  |
|  |  |  |  | c.12721G>A | p.Ala4241Thr | Missense |  | Heterozygous | 0.000486 |  |
|  |  |  |  | c.6962_6968del | p.His2321Profs*4 | Frameshift | North Africa | Homozygous | 0.000033 |  |
|  |  |  | Low motility (17%)  Absence of CP(9+0)  A missing or irregular arrangement of ODFs and peripheral microtubule doublets  In the end piece, the atypical “5 + 2” structure was frequently observed | c.378_379del | p.Lys127Arg fs*5 | Frameshift | China | Homozygous | NA | 32681648 |
|  |  |  | Very low progressive motility (0, 0.5%)  The absent, short, and coiled flagella were the main defections  Absence of CP(9+0) (main defect)  MS were fragmentary with randomly organized mitochondria | c.2781+1G>T | - | Splicing variant | China | Heterozygous | NA | 33704367 |
|  |  |  |  | c.10348delT | p.Fhe3459fs | Frameshift |  | Heterozygous | NA |  |
|  |  |  |  | c.6178G>A | p.Ala2060Thr | Missense |  | Heterozygous | NA |  |
|  |  |  |  | c.13748G>T | p.Arg4583Leu | Missense |  | Heterozygous | 0.000198 |  |
|  |  | *DNAH10* | Very low sperm concentration (1.6,0.5,6.6×10^6^/mL) except patients harboring the compound heterozygous mutation c.12235del and c.7260dup (71.2,72.6×10^6^/mL)  Very low progressive motility (<3 %)  Coiled flagella were most frequently observed  Absence of IDAs(main defect)  Disorganized peripheral microtubule doublets, MS, ODFs, and FS, as well as absent CP. | c.12838G>A | p.Gly4280Arg | Missense | China | Homozygous | NA | 34237282 |
|  |  |  |  | c.7601C>T | p.Thr2534Met | Missense |  | Homozygous | NA |  |
|  |  |  |  | c.5663G>A | p.Arg1888Gln | Missense |  | Heterozygous | NA |  |
|  |  |  |  | c.11887C>T | p.Arg3963Cys | Missense |  | Heterozygous | NA |  |
|  |  |  |  | c.12235del | p.Ser4079Alafs*5 | Frameshift |  | Heterozygous | NA |  |
|  |  |  |  | c.7260dup | p.Glu2421Argfs*26 | Frameshift |  | Heterozygous | NA |  |
|  |  |  | Very low motility (6.3%; 13.3%) and progressive motility (3.8%; 2.4%)  Coiled and short flagella were most frequently observed  With quite a few normal flagella (25.3,23.0)  Defects in IDAs (main defect) | c.2514delG | p.L839* | Frameshift | China | Homozygous | NA | 34657236 |
|  |  |  |  | c.10820 T > C | p.M3607T | Missense |  | Heterozygous | NA |  |
|  |  |  |  | c.12692C > T | p.T4231I | Missense |  | Heterozygous | NA |  |
|  |  | *DNAH17* | Very low progressive motility (<5 %)  All but one individual (harboring the mutation c.[10496C>T;10784T>C]) showed a severe reduction of the number of spermatozoa with normal morphology  Absence of the ODAs in the sperm cells only (main defect)  Absence of CP(9+0), peripheral microtubule doublets and ODFs | c.1293_1294del | p.Tyr431* | Nonsense. | France/Italy | Heterozygous | 0.000016 | 31178125 |
|  |  |  |  | c .7994_8012del | p.Gly2665-Glufs* | Frameshift |  | Heterozygous | NA |  |
|  |  |  |  | c.5486G>A | p.Cys1829Tyr | Missense | Algeria | Homozygous | NA |  |
|  |  |  |  | c.[10496C>T;10784T>C] | [Pro3499Leu;Leu3595Pro] | Missense | Morroco | Homozygous | NA |  |
|  |  |  |  | c.10486_10497dup | p.Val3496_Pro3499dup | Copy number variant | France | Homozygous | NA |  |
|  |  |  | Low sperm concentration (<15×10^6^/mL)  Very low progressive motility (<1.5 %)  Short, bent, or absent flagella were most frequently observed  Absence of ODAs surrounded by a disorganized MS or FS | c.4445C > T | p.Ala 1482Val | Missense | China | Heterozygous | 0.00003329 | 31841227 |
|  |  |  |  | c.6857C > T | p.Ser2286Leu | Missense |  | Heterozygous | 0.0004 |  |
|  |  |  | Very low progressive motility (<5 %)  Short, coiled and bent flagella were most frequently observed  Absence of CP, peripheral microtubule doublets and ODAs | c.6308C>T | p.Ala2103Val | Missense | Pakistan | Homozygous | 8.0x 10^-6^ | 33070343 |
|  |  |  |  | c.11803C>T | p.Gln3935* | Nonsense |  | Homozygous | NA |  |
|  |  |  |  | c.5707C>T | p.Arg1903Cys | Missense | China | Homozygous | NA |  |
|  |  |  | Low sperm concentration (10×10^6^/mL)  Very low motility (5%)  Absence of CP and peripheral microtubule doublets Thickened MS, disorganized ODFs  In the end piece, the “5 + 2” structure was frequently observed | c. 4810C>T | p.Arg1604Cys | Missense | China | Homozygous | 0.000025 | 34373205 |
|  |  |  | The percentage of morphologically normal sperm fell within the normal range (75~85%)  Low sperm motility(≤25.0%) and low progressive motility (≤17.5% )  A frequent absence of peripheral microtubule doublets 4–7, with absence of ODFs  ODA was clearly observed attached to each of the nine peripheral microtubule doublets | c.5408G>A | p.Cys1803Try | Missense | Pakistan | Homozygous | NA | 31658987 |
|  |  |  | Very low progressive motility (12.8, 0.2%)  Significantly increased frequency of MTDs 4-7  missing at the principal piece and end piece  Absence of the CP and peripheral microtubule doublets and disarranged ODFs | c.12915+1C > T | - | Splicing variant | China | Heterozygous | NA | 33423959 |
|  |  |  |  | c.13202G > A | p.Pro4401Leu | Missense |  | Heterozygous | NA |  |
|  |  |  |  | c.8512-2A > G | - | Splicing variant |  | Heterozygous | NA |  |
|  |  |  |  | c.13294C > T | p.Arg4432Cys | Missense |  | Heterozygous | NA |  |
|  |  |  | Low sperm concentration (14×10^6^ /mL)  Very low progressive motility (9.4%)  Extremely low percent of sperm with normal morphology (1.5%) while quite a few normal flagella could be observed（69%） | c.5368C > T | p.Arg1790Cys | Missense | China | Heterozygous | NA | 35932098 |
|  |  |  |  | c.13183C > T | p.Arg4395Trp | Missense |  | Heterozygous | NA |  |
|  |  |  | Low motility (≤17%)  Morphological analysis of the spermatozoa revealed normal structure | c.1048 C > T | p.Arg350* | Nonsense | China | Heterozygous | 0.000013 | 34126833 |
|  |  |  |  | c.3390G > A | p.Met1130Ile | Missense |  | Heterozygous | 0.000017 |  |
|  |  | *WDR63* | The sperm counts were dramatically lower than the normal reference values(12, 0×106)  No motile sperm was observed | c.163 C > T | p.Arg55* | Nonsense | China | Homozygous | 0.000033 | 34782613 |
|  |  |  |  | c.1075 C > T | p.Arg359* | Nonsense |  | Homozygous | 0.000091 |  |
|  |  | *CFAP43* | No motile sperm was observed  Short flagella were most frequently observed | c.3661-2delA | - | Splicing variant | China | Homozygous | NA | 29277146 |
|  |  |  | No motile sperm was observed  Short flagella were most frequently observed  Severe disorganization of the FS, ODFs, and axonemal disassembly (main defect)  Absence of CP(9+0) | c.899_900del | p.Arg300Lysfs*22 | Frameshift | Pakistan | Heterozygous | 8.0x 10^-6^ | 34100391 |
|  |  |  |  | c.1577_1578del | p.Thr526Serfs*43 | Frameshift |  | Heterozygous | 0.000025 |  |
|  |  |  |  | c.1577_1578del | p.Thr526Serfs*43 | Frameshift |  | Homozygous | 0.000025 |  |
|  |  |  | No motile sperm was observed  Short and coiled flagella were most frequently observed  Absence of CP(9+0) (main defect) | c.1140_1143del | p.Asn380Lys­fs*3 | Frameshift | China | Homozygous | 8.0x 10^-6^ | 30904354 |
|  |  |  |  | c.739A>T | p.Lys247* | Nonsense |  | Homozygous | NA |  |
|  |  |  |  | c.1474G>C | p.Gln492Arg | Missense |  | Homozygous | NA |  |
|  |  |  |  | c.4600C>G | p.Leu1534Val | Missense |  | Homozygous | 0.000016 |  |
|  |  |  | No spermatozoa with progressive motility could be  observed  Short, coiled, and absent flagella were the most frequently observed.  Absence of CP(9+0) (main defect)  Hypertrophy and hyperplasia of FS, disorganization in other axonemal and periaxonemal structures | c.2802T>A | p.Cys934* | Nonsense | China | Heterozygous | 8.0x 10^-6^ | 28552195 |
|  |  |  |  | c.4132C>T | p.Arg1378* | Nonsense |  | Heterozygous | 0.000013 |  |
|  |  |  |  | c.253C>T | p.Arg85Trp | Missense |  | Heterozygous | 0.000033 |  |
|  |  |  |  | c.3945_4431del | p.Ile1316Leufs*10 | Frameshift |  | Heterozygous | NA |  |
|  |  |  |  | c.386C>A | p.Ser129Tyr | Missense |  | Heterozygous | NA |  |
|  |  |  | No motile sperm was observed  Short and coiled flagella were most frequently observed  Absence of CP(9+0) (main defect)  CP, when present, was misoriented compared to control sections in which the CP is normally parallel to the axis of the two longitudinal columns of the FS  Severe axonemal and periaxonemal defects affecting the ODF, the FS, and the MS, | c.2141+5G>A | p.Lys714Val*11 | Splicing variant | Turkey | Homozygous | NA | 29449551 |
|  |  |  |  | c.1240_1241delGT | p.Val414Leufs*46 | Frameshift | Afghanistan ranian | Homozygous | 0.000033 |  |
|  |  |  |  | c.2658G>A | p.Trp886* | Nonsense | Algeria | Homozygous | 0.000099 |  |
|  |  |  |  | c.2680C>T | p.Arg894* | Nonsense | Algeria | Homozygous | 8.0x 10^-6^ |  |
|  |  |  |  | c.3882delA | p.Glu1294Aspfs*47 | Frameshift | Tunis | Homozygous | NA |  |
|  |  |  |  | c.3374delT | p.Gly1125Glufs*12 | Frameshift | Iran | Homozygous | 8.0x 10^-6^ |  |
|  |  |  |  | c.3352C>T | p.Arg1118* | Nonsense | Tunis | Homozygous | 0.0000033 |  |
|  |  |  |  | c.1302dupT | p.Leu435Serfs*26 | Frameshift | France | Heterozygous | NA |  |
|  |  |  |  | c.1040T>C | p.Val347Ala | Missense |  | Heterozygous | 0.000074 |  |
|  |  |  |  | c.3541-2A>C | p.Ser1181Lysfs*4 | Frameshift | Tunis | Homozygous | NA |  |
|  |  | *CFAP44* | Very low progressive motility (≤0.5%)  Short flagella were most frequently observed | c.2935_2944del | p.Asp979* | Frameshift | China | Homozygous | NA | 29277146 |
|  |  |  |  | c.2935_2944del | p.Asp979* | Frameshift |  | Homozygous | NA |  |
|  |  |  |  | c. 1769 T >A | p.Leu590Gln | Missense |  | Homozygous | 8.0x 10^-6^ |  |
|  |  |  |  | c.2005_2006del | p.Met669Val fs*13 | Frameshift |  | Homozygous | 8.0x 10^-6^ |  |
|  |  |  |  | c. 3262 G >A | p.Gly1088Ser | Missense |  | Heterozygous | NA |  |
|  |  |  |  | c. 1718 C >A | p.Pro573His | Missense |  | Heterozygous | NA |  |
|  |  |  | No motile sperm was observed  Short and coiled flagella were most frequently observed  Absence of CP(9+0) (main defect) | c.4963C>T | p.Arg1655* | Nonsense | China | Homozygous | 0.000128 | 30904354 |
|  |  |  | No spermatozoa with progressive motility could be  observed  Short, coiled, and absent flagella were the most frequently observed.  Absence of CP(9+0) (main defect)  Hypertrophy and hyperplasia of FS, disorganization in other axonemal and periaxonemal structures | c.2005_2006delAT | p.Met669Valfs*13 | Frameshift | China | Homozygous | 8.0x 10^-6^ | 28552195 |
|  |  |  | No motile sperm was observed  Short and coiled flagella were most frequently observed  Absence of CP(9+0) (main defect)  A single central microtubule (9+1 conformation) were observed in about 10% of cases  Severe axonemal and peri-axonemal defects affecting the ODF, the FS, and the MS, | c.1890+1G>A | p.Pro631Ile*22 | Splicing variant | Tunis | Homozygous | NA | 29449551 |
|  |  |  |  | c.3175C>T | p.Arg1059* | Nonsense | Tunis | Homozygous | NA |  |
|  |  |  |  | c.2818dupG | p.Glu940Glyfs*19 | Frameshift | Morocco | Homozygous | NA |  |
|  |  |  |  | c.1387G>T | p.Glu463* | Nonsense | Algeria | Homozygous | NA |  |
|  |  |  |  | c.4767delT | p.Ile1589Metfs*6 | Frameshift | Algeria | Homozygous | NA |  |
|  |  | *CCDC39* | No motile sperm was observed  Absent flagella were most frequently observed  Absence of CP(9+0) (main defect)  Partially absence of CP and disorganized peripheral microtubule doublets | c.983 T>C | p. Leu328Pro | Missense | China | Homozygous | NA | 34674941 |
|  |  |  | No motile sperm was observed  Low sperm concentration (1.4×10^6^/mL)  Coiled flagella were most frequently observed | c.732_733del | p.Ala245PhefsTer18 | Frameshift | China | Heterozygous | NA | 35795318 |
|  |  |  |  | c.2800_2802dup | p.Val934dup | Nonframeshift duplication |  | Heterozygous | 0.000068 |  |
|  |  | *CCDC40* | No motile sperm was observed  Coiled flagella were most frequently observed  Absence of IDAs | c.901C>T | p.Arg301* | Nonsense | China | Heterozygous | 0.000017 | 35449766 |
|  |  |  |  | c.2065_2068dup | p.Ala690Glyfs*67 | Frameshift |  | Heterozygous | NA |  |
|  | Radial Spokes | *CFAP61* | Low motility (<40%) and progressive motility (<32%)  Missing of peripheral microtubule doublets, dynein  arms, RSs, and/or CP  Loss of Calmodulin- and spoke-associated complex protein | c.451_452del | p. Ile151Asn fs*13 | Frameshift | Pakistan | Homozygous | NA | 35174165 |
|  |  |  |  | c.847C > T | p. Arg283* | Nonsense |  | Homozygous | NA |  |
|  |  |  | Low progressive motility (20%)  36% bent, 18% with no tail, 12% with a short tail, 50% with an irregular shape and 30% with a coiled tail. | c.143+5G>A | - | Splicing variant | China | Homozygous | 0.00016 | 34792097 |
|  |  |  | Low sperm concentration and motility  Very low progressive motility  Irregular calibers, coiled, short, and absent flagella  Absence of CP, the loss of peripheral microtubule doublets, abnormal ODFs and defective MS | c.1654C>T | p.Arg552Cys | Missense | China | Heterozygous | 0.000099 | 35387802 |
|  |  |  |  | c.2911G>A | p.Asp971Asn | Missense |  | Heterozygous | 0.000132 |  |
|  |  |  |  | c.144–2A>G | - | Splicing variant |  | Heterozygous | NA |  |
|  |  |  |  | c.1666G>A | p.Gly556Arg | Missense |  | Heterozygous | 0.000239 |  |
|  |  | *CFAP91/ MAATS1* | Very low motility (0-10%)  Absence of CP(9+0) (main defect)  Head malformations were also observed  Peri axonemal structural defects such as an abnormal number of ODFs | c.682+1G>A | - | Splicing variant | Tunis | Homozygous | 8.0x 10^-6^ | 32161152 |
|  |  |  |  | c.124G>C | p.Asp42His | Missense |  | Homozygous | 0.000082 |  |
|  |  | *CFAP206* | Very low sperm concentration (0.2×10^6^/mL)  No motile sperm was observed  Short flagella were most frequently observed  RS and Calmodulin- and spoke-associated complex were strongly disorganized | c.1430dupA | p.Asn477Lysfs*15 | Frameshift | Iran | Homozygous | NA | 34255152 |
|  |  | *CFAP251* | A total absence of sperm motility was observed  Short and irregular caliber flagella were most frequently observed  A high rate (56%–95%) of acrosomal abnormalities was also observed  Completely disorganized axonemal and peri axonemal defects affecting the ODFs, FS, and MS | c.2862+1G>A | - | Splicing variant | Iran | Homozygous | NA | 30122540 |
|  |  |  |  | c.3007_3337del | p.Ile1003Lysfs26 | Frameshift | Tunis | Homozygous | NA |  |
|  |  |  | Very low total and progressive motility (<1%)  Absent and coiled flagella were most frequently observed  Severe disorganization in axonemal and other peri axonemal structures | c.1192-3C>G | - | Splicing variant | China | Homozygous | NA | 35087568 |
|  |  |  | Very low progressive motility (0 , 0.2, 1.5%)  Short, absent, and coiled flagella were most frequently observed | c.799 C > T | p.Arg267* | Nonsense | China | Homozygous | 8.0x 10^-6^ | 30310178 |
|  |  |  |  | c.415 C > T | p.Gln139* | Nonsense |  | Heterozygous | NA |  |
|  |  |  |  | c.1718delT | p.Phe574Leu*3 | Frameshift |  | Heterozygous | NA |  |
|  |  |  |  | c.1286 + 2 T > C | - | Splicing variant |  | Homozygous | NA |  |
|  |  |  | No motile sperm was observed  Short and absent flagella were most frequently observed  A short MS with very low mitochondrial density was observed | c.123delA | p.Asp42Metfs*4 | Frameshift | Lebanon | Homozygous | 8.0x 10^-6^ | 30122541 |
|  |  |  |  | c.331G>T | p.Glu111* | Nonsense | France | Heterozygous | 0.001158 |  |
|  |  |  |  | c.1588_1589delCT | p.Leu530Valfs*4 | Frameshift |  | Heterozygous | NA |  |
|  | N-DRC | *DRC1* | Low sperm concentration (11.3, 8.84×10^6^/mL)  Very low progressive motility (0, 0.5%)  Disordered structure of the flagellar axoneme, with microtubules being scattered in the cytoplasm and with normal centriole  implantation and implantation nest formation | c. 1660C>T | p.Arg554* | Nonsense | China | Homozygous | NA | 34169321 |
|  |  |  |  | c.238C>T | p.Arg80* | Nonsense |  | Homozygous | 0.000025 |  |
|  |  |  | Low sperm concentration (5.3×10^6^/mL)  No motile sperm was observed  Cytoplasmic masses in the tails  Disorganized microtubules | c.1296 G>A | p.Trp432* | Nonsense | China | Homozygous | NA | 34815526 |
|  |  |  | No motile sperm was observed  Coiled flagella were most frequently observed | c.156-1724_244-2550del | p. Glu53Asnfs*13 | Copy number variant | China | Homozygous | NA | 35873463 |
|  | ODF | *ODF2* | No motile sperm was observed  Only 2.0% of the sperm were alive  Short flagella were most frequently observed  Loss of ODFs(main defect)  Absence of CP(9+0) | c.242 A>G | p.Lys81Arg | Missense | China | Heterozygous | NA | 35102900 |
|  |  | *CFAP58* | Very low progressive motility (1.5, 0%)  Short, absent, and irregular caliber flagella were most frequently observed  Inadequate doublet microtubules and the CPs were absent | c.323C>T | p.Ser108Leu | Missense | China | Heterozygous | NA | 33314088 |
|  |  |  |  | c.1855C>T | p.Arg619Trp | Missense |  | Heterozygous | 0.000362 |  |
|  |  |  |  | c.1883A>G | p.Tyr628Cys | Missense |  | Heterozygous | NA |  |
|  |  |  |  | c.2020G>T | p.Ala674Ser | Missense |  | Heterozygous | NA |  |
|  |  |  | Very low total (0-6.4%) and progressive motility (0-2.1%)  Coiled and short flagella were most frequently observed  Absence of CP (9+ 0 or 9+1), absence of peripheral microtubule doublets (main defect)  Disorganization of CP, peripheral microtubule doublets, nexin, dynein arms, MS, and/or ODFs  The number of ODFs was almost doubled in some of the flagellar mid-piece | c.2092C>T | p.Arg698* | Nonsense | China | Homozygous | 0.000058 | 32791035 |
|  |  |  |  | c.1429del | p.lle477* | Frameshift |  | Heterozygous | 8.0x 10^-6^ |  |
|  |  |  |  | c.2092C>T | p.Arg698* | Nonsense |  | Heterozygous | 0.000058 |  |
|  |  |  |  | c.2052del | p.His685Thrfs*7 | Frameshift |  | Homozygous | NA |  |
|  |  |  |  | c.1696C>T | p.Gln566* | Nonsense |  | Homozygous | NA |  |
|  |  |  |  | c.2274C>A | p.Tyr758* | Nonsense |  | Homozygous | NA |  |
|  | FS | *FSIP2* | Low sperm motility (6.50 ±1.32%)  Absence of CP(9+0) (main defect)  Disorganization of peripheral microtubule doublets | c.16246_16247insCCCAAATATCACC | p. Thr5416fs*7 | Frameshift | China | Heterozygous | NA | 33631238 |
|  |  |  |  | c.17323C > T | p.Gln5774* | Nonsense |  | Heterozygous | NA |  |
|  |  |  | Very low sperm concentration except the patient harboring the homozygous mutation on c.16389_16392delAAT  Very low sperm motility (≤5%)  Short and irregular caliber flagella were most frequently observed  Completely disorganized and dysplastic of FS  Total absence of MS  ODFs 3 and 8 were not replaced by the longitudinal columns in some principal-piece  Absence and disorganization of CP  Some severe nuclear alterations, particularly in chromatin texture | c.910delC | p.Gln304Lysfs*13 | Frameshift | Tunis | Homozygous | NA | 30137358 |
|  |  |  |  | c.[1606_1607insTGT; 1607_1616delAAAGATTGCA] | p.Lys536Metfs*1 | Frameshift |  | Homozygous | NA |  |
|  |  |  |  | c.2282dupA | p.Asn761Lysfs*4 | Frameshift |  | Homozygous | NA |  |
|  |  |  |  | c.3252delA | p.Lys1084Asnfs*19 | Frameshift |  | Homozygous | NA |  |
|  |  |  |  | c.8003C>A | p.Ser2668* | Nonsense |  | Homozygous | NA |  |
|  |  |  |  | c.16389_16392delAAT | p.Glu5463Glufs*7 | Frameshift |  | Homozygous | NA |  |
|  |  |  | Low total (18%) and progressive motility (16%)  Large proportion sperms (18.1%) showed thicker neck and midpiece (main defect)  Thick midpiece and abnormal mitochondrial arrangement  Excessive residual cytoplasm was visible leading to enlargement neck  FS was dysplasia with two layers and shortened  significantly in the principle piece | c.1494C > A | p.Cys498* | Nonsense | China | Heterozygous | NA | 35672654 |
|  |  |  |  | c.11020_11024del | p.Tyr3675Cysfs*3 | Frameshift |  | Heterozygous | 0.000112 |  |
|  |  |  | Low total (8.9-37.17%) and progressive motility (1.2-8.63%)  Short tails, thin principal piece and abnormal mid piece  MS was dysplastic and disorganized, and FS was absent | c.8368_8369insC | p.2790fs | Frameshift | China  China | Homozygous | NA | 34125190 |
|  |  |  | Low total (5.9, 7.1%) and progressive motility (3.7, 6.1%)  Short flagella were most frequently observed  Abnormal heads (flat, round, triangular-like) were also detected | c.1750T>A | p.Cys584Ser | Missense | China | Heterozygous | NA | 34935173 |
|  |  |  |  | c.13600A>G | p.Ile4534Val | Missense |  | Heterozygous | NA |  |
|  |  |  | Low total (21.9, 17.3%) and progressive motility (6.5, 9.8%)  Abnormal sperm neck and short flagella were most frequently observed  The sperm midpiece was thick with redundant residual body.  Extra disassembled peripheral microtubule doublets and ODFs were observed | c. 1907C > A | p.Ser636* | Nonsense | China | Homozygous | NA | 30745215 |
|  |  |  |  | c.8030_8031insA | p.Thr2680Asnfs*9 | Frameshift |  | Homozygous | 0.00022 |  |
|  |  |  | >70% of round heads  Severe disorganized arrangement or absence of the peri axoneme and ‘9+2’ axoneme structure  Some heads of spermatozoa were almost spherical with absent or minute acrosome, and others depicted irregular ovoid and possessed hypoplastic acrosome, which were either not well attached to the nuclear membrane or were completely disengaged.  Chromatin compaction was low, and nuclear pseudo vacuoles | c.19981C>T | p.Arg6661* | Nonsense | China | Homozygous | NA | 35654582 |
|  |  |  |  | c.18448G>A | p.Val6150Ile | Missense |  | Heterozygous | 0.000066 |  |
|  |  |  |  | c.5238_5240del | p.Glu1746del | Frameshift |  | Heterozygous | NA |  |
|  |  |  |  | c.5480A>T | p.Asp1827Val | Missense |  | Heterozygous | NA |  |
|  |  |  |  | c.9056T>C | p.Ile3019Thr | Missense |  | Heterozygous | NA |  |
|  |  |  |  | c.10823T>C | p.Ser5933Phe | Missense |  | Heterozygous | NA |  |
|  |  |  |  | c.17798C>T | p.Leu3608Ser | Missense |  | Heterozygous | 0.000497 |  |
|  |  | *AKAP3* | Low sperm motility  Absence or misarrange of peripheral or central microtubules  Slightly higher rate of abnormal acrosomal morphology | c.2286_2287del | p.His762Glnfs*22 | Frameshift | China | Homozygous | NA | 35228300 |
|  |  |  |  | c.44G>A | p.Cys15Tyr | Missense |  | Homozygous | 0.000412 |  |
|  |  | *AKAP4* | Low sperm concentration (2.1, 1.5, 2.6×10^6^/mL)  Low total (26.3, 17.9, 21.5%) and progressive motility (8.4, 4.2, 11.3%)  Coiled and bent flagella were most frequently observed  abnormalities of FS and absence of double microtubule(main defect) | c. 1285 C >T | p.Arg429Cys | Missense | China | Hemizygous | 0.000033 | 34415320 |
|  | Centrosome | *CEP135* | Very low sperm motility (1.8%)  Very low sperm vitality (2.9%)  Coiled and absent flagella were most frequently observed | c. 1364A>T | p.Asp455Val | Missense | China | Homozygous | NA | 28866084 |
|  |  | *DZIP1* | Low sperm concentration (<6×10^6^/mL)  No spermatozoa with progressive motility  Absent flagella accounted for more than 90% spermatozoa  Absent or very short axoneme | c.188G>A | p.Arg63Gln | Missense | China | Homozygous | NA | 32051257 |
|  |  |  |  | c.690T>G | p.Tyr230* | Nonsense | China | Homozygous | NA |  |
| Genes encoding proteins interacting with other proteins to participate in flagellar assembly | | *TTC21A/IFT39A* | 2/3 with low sperm concentration (13.9, 9.4×10^6^/mL)  Very low total (0.8, 6.7, 1.0%) and progressive motility (0, 1.7, 0%)  Abnormal necks and short flagella were the most frequently observed  Cytoplasm residue and scattered and disorganized axonemal components  Absent or misplaced central-pair microtubules, absent or misplaced  peripheral microtubule doublets, or hyperplasia of the fibrous sheaths | c.716+1G>A | p.Ile240* | Splicing variant | China | Homozygous | NA | 30929735 |
|  |  |  |  | c.341A>G | p.Tyr114Cys | Missense |  | Heterozygous | 8.0x 10^-6^ |  |
|  |  |  |  | c.2329C>T | p.Gln777* | Nonsense |  | Heterozygous | NA |  |
|  |  |  |  | c.2563del | p.Val855* | Nonsense |  | Homozygous | NA |  |
|  |  |  |  | c.3116+5G>T | - | Splicing variant | Tunis | Homozygous | 0.000487 |  |
|  |  |  | Very low motility (4.32%) or progressive motility (1.85%)  Flagella with irregular caliber were the most frequently observed  Some spermatozoa heads were significantly larger, cytoplasmic residues remained in the flagella, absent and misplaced CP and peripheral doublet microtubules | c.3450+2delT | - | Splicing variant | China | Homozygous | NA | 35920310 |
|  |  | *TTC29* | Very low total motility (1.3,0, 0.8%) and no spermatozoa with progressive motility  The patient with the early truncating mutation of *TTC29* (c.412_425del [p.Asp138Leufs*10]) had a higher malformation rate of sperm flagella than those of the other two subjects with late truncating mutations of *TTC29*  Adramatic disorganization in axonemal or other peri axonemal structures, such as, the unassembled ODFs and the absent (9 + 0)or misplaced CP | c.1107C>G | p.Tyr369* | Nonsense | China | Homozygous | NA | 31735294 |
|  |  |  |  | c.412_425del | p.Asp138Leufs*10 | Frameshift |  | Homozygous | NA |  |
|  |  |  |  | c.1107C>G | p.Tyr369* | Nonsense |  | Heterozygous | NA |  |
|  |  |  |  | c.977+1G>T | p.Ser326Profs*8 | Frameshift |  | Heterozygous | NA |  |
|  |  |  | One patient with mutation of TTC29(c.176+1G>A) had a low sperm count (16.8×10^6^)  Very low motility (0-10%) or progressive motility (0-2%)  Abnormal midpiece and FS together with severe axonemal disorganization  Abnormal axonemal structure including lacking the CP(9+0) and a few sections displaying global microtubule doublets disorganization  The percentage of sections with axonemal defects observed (36%)was much lower than what was previously reported for MMAF-affected individuals carrying mutations in *CFAP43* and *CFAP44*(95% in average) | c.176+1G>A | p.Tyr60* | Nonsense | Africa | Homozygous | 0.000158 | 31735292 |
|  |  |  |  | c.330_334delGGAGG | p.Glu111Alafs* | Frameshift | Iran | Homozygous | 0.000017 |  |
|  |  |  |  | c.750C>A | p.Tyr250* | Nonsense | Iran | Homozygous | NA |  |
|  |  | *IFT144/WDR19* | No spermatozoa with progressive motility  Coiled flagella were the most frequently observed  13.5% of the sperm cells were found to have normal flagella  A mostly vacuolar cross-sectional phenotype, damaged axoneme, and absence of microtubule structures, with only small amounts of debris-like substances (main defect)  Absence of CP (9+0) | c. 3811A>G | p.Lys1271Gln | Missense | China | Homozygous | NA | 32323121 |
|  |  | *IFT74* | Individual IFT74_1:Very low progressive motility  Short flagella were the most frequently observed (16%). The pattern of the other abnormalities showed no specificity  Abnormal MS and cytoplasmic bags containing unassembled flagellar components, 27% of abnormal axonemal structure, all characterized by the absence of some peripheral doublets  Individual IFT74_2: a low sperm count (7 ×10^6^/ml), no motile and morphologically normal sperm, low vitality (22%)  Irregular caliber (84%) and/or a short (73%) flagella were the most frequently observed | c.256G > A | p.Gly86Ser | Missense | Tunis/Algeria | Homozygous | NA | 33689014 |
|  |  | *ARMC2* | Low sperm concentration( 3.0, 8.0 ×10^6^/mL)  No motile sperm was observed  Coiled and short flagella were the most frequently observed  Absence of CP(9+0)(main defect)  Disorganizations of axonemal structure and the peri-axonemal structures were also observed, such as ODFs | c.182C>G | p.Ser61* | Nonsense | Pakistan | Homozygous  Homozygous | NA | 34493464 |
|  |  |  | Low sperm motility (≤6%)  Short flagella were the most frequently observed  Absence of CP(9+0)(main defect)  A dramatic axonemal disorganization associated with peri axonemal structural defects such as unassembled ODFs  Truncated flagella or the presence of cytoplasmic structures encompassing unassembled axonemal components | c.1023+1G>A | p.Glu283Ala fs*2 | Frameshift | Tunis | Homozygous  Homozygous | NA | 30686508 |
|  |  |  |  | c.2279T>A | p.Ile760Asn | Missense | Tunis | Homozygous  Homozygous | NA |  |
|  |  |  |  | c.2353_2354delTT | p.Leu785Metfs*5 | Frameshift | Tunis | Homozygous  Homozygous | NA |  |
|  |  |  |  | c.1284_1288delACAAA | p.Lys428Asn fs*3 | Frameshift | Iran | Homozygous  Homozygous | NA |  |
|  |  |  |  | c.421C>T | p.Gln141* | Nonsense | China | Homozygous | NA |  |
|  |  |  | Very low sperm concentration (0.9-1.4×10^6^/mL)  Very low motility (0-4.2%) or progressive motility (0-3.2%)  Absence of CP(9+0)(main defect)  Normal length and ultrastructure sperm existed | c.1264C > T | p.Arg422* | Nonsense | China | Homozygous | NA | 35543806 |
|  |  | *CCDC34* | Very low sperm concentrations, total and progressive motility  Short and absent flagella were the most frequently observed  Disordered ODFs and abnormal ‘9+2’ microtubule structures | c.731dup | p.Asn244Lysfs*3 | Frameshift | China | Homozygous | 0.000017 | 34348960 |
|  |  |  |  | c.799_817del | p.Glu267Lysfs*72 | Frameshift |  | Homozygous | NA |  |
|  |  | *DNHD1/CCDC35* | The progressive motility rate in three of these eight affected individuals decreased dramatically to zero, and the other five had progressive motility rates of 0.5%, 0.8%, 3.2%, 6.3%, and 16.5%  Absence of CP (9+0), axoneme disorganization and absence of peripheral doublets (main defect 95%)  A swollen mid-piece with poorly assembled MS, a cytoplasmic  mass containing different components, loosely assembled MS, and a completely disorganized mid-piece with a misshapen MS | c.5560C>T | p.Arg1854Cys | Missense | China | Heterozygous | NA | 34932939 |
|  |  |  |  | c.6498T>G | p.Tyr2166∗ | Nonsense |  | Heterozygous | NA |  |
|  |  |  |  | c.8782C>T | p.Arg2928∗ | Nonsense |  | Homozygous | 0.000434 |  |
|  |  |  |  | c.522_525del | p.Arg174Serfs∗3 | Frameshift |  | Heterozygous | 8.0x 10^-6^ |  |
|  |  |  |  | c.911G>A | p.Arg304Gln | Missense |  | Heterozygous | 0.000124 |  |
|  |  |  |  | c.425_428del | p.Asp142Valfs∗35 | Frameshift |  | Heterozygous | NA |  |
|  |  |  |  | c.4072C>T | p.Arg1358Cys | Missense |  | Heterozygous | 0.000704 |  |
|  |  |  |  | c.8909A>G | p.Tyr2970Cys | Missense |  | Homozygous | 0.000042 |  |
|  |  |  |  | c. 9649C>T | p.Arg3217∗ | Nonsense |  | Heterozygous | 0.000043 |  |
|  |  |  |  | c.12473A>G | p.His4158Arg | Missense |  | Heterozygous | NA |  |
|  |  |  |  | c.12453G>A | p.Trp4151∗ | Nonsense |  | Heterozygous | NA |  |
|  |  |  |  | c.4141C>T | p.Gln1381∗ | Nonsense |  | Heterozygous | NA |  |
|  |  |  |  | c.14234T>C | p.Val4745Ala | Missense |  | Heterozygous | 0.001041 |  |
|  |  |  |  | c.5347delC | p.Gln1783Serfs∗28 | Frameshift |  | Heterozygous | 0.000029 |  |
|  |  | *QRICH2* | No progressive motility of spermatozoa was observed  Absence of CP (9+0) (main defect)  Patient with mutation *QRICH2*(c.192G>A):  Integrated and regularly arranged peripheral microtubule doublets and ODF in the mid piece. In the principal piece, some ODF and peripheral microtubule doublets were absent, the remainders were disorganized  Patient with mutation *QRICH2*(c.3037C>T):  The atypical 6 + 0 composition of axonemal microtubules in the mid piece, the irregular arrangement of the ODF and peripheral microtubule doublets in the principal piece. | c.192 G > A | p. Trp64* | Nonsense | China | Homozygous | NA | 30683861 |
|  |  |  |  | c.3037C>T | p.Arg1013* | Nonsense |  | Homozygous | 0.000025 |  |
|  |  |  | Very low total (7,0%) and progressive (2, 0%) motility | c.3501C>G | p.Tyr1167* | Nonsense | North Africa | Homozygous | NA | 31292949 |
|  |  |  |  | c.4614C>G | p.Tyr1538* | Nonsense |  | Homozygous | 8.0x 10^-6^ |  |
| Genes of which encoding proteins ‘location and function were undefined or questionable | | *AK7* | A moderate decrease in sperm vitality  Very low progressive (1, 0%) motility  A severe disorganization of the axonemal structure; in particular, abnormal midpiece and dysplasia of the fibrous sheath  were observed, associated with the lack of CP and disorganization  of the peripheral doublets | c.2018T > G | p.Leu673Pro | Missense | Europe | Homozygous | NA | 29365104 |
|  |  |  | Low sperm concentrations (8.3, 8.8×10^6^/mL), total motility(4.9, 4.0%)  Coiled and short flagella were the most frequently observed | c.1846G > A | p.Glu616Lys | Missense | China | Homozygous | 0.000222 | 34854019 |
|  |  | *CFAP47* | Low total (10.0-23.3%) and progressive (5.0-18.5%) Disorganization of ODFs, and absence of peripheral or central microtubules at the midpiece and principal piece  The deficiency of SPAG16 (a component of core axoneme complex) was revealed | c.7154T>A | p.Ile2385Asn | Missense | China | Hemizygous | NA | 33472045 |
|  |  |  |  | c.5224A>G | p.Ser1742Gly | Missense |  | Hemizygous | NA |  |
|  |  |  |  | c.8668C>A | p.Pro2890Thr | Missense |  | Hemizygous | NA |  |
|  |  |  |  | GRCh37/hg19.Xp21.1x0 | Eliminating the entire CFAP47 | Segment deletion | Australia | Hemizygous | NA |  |
|  |  | *CFAP65* | No spermatozoa with progressive motility  Absent and short flagella were most frequently observed (80%)  Absence of CP (9+0) (main defect)  Hypertrophy and hyperplasia of fibrous sheaths | c.1775delC | p.Pro592Leufs*8 | Frameshift | China | Homozygous | **NA** | 31501240 |
|  |  |  |  | c.3072_3079dup | p.Arg1027Profs*41 | Frameshift |  | Heterozygous | NA |  |
|  |  |  |  | c.1946delC | p.Pro649Argfs*5 | Frameshift |  | Heterozygous | NA |  |
|  |  |  |  | c.1580delT | p.Leu527Argfs*31 | Frameshift |  | Homozygous | NA |  |
|  |  |  |  | c.4855C >T | p.Arg1619* | Nonsense |  | Heterozygous | 8.0x 10^-6^ |  |
|  |  |  |  | c.5270T>A | p.Leu1757* | Nonsense |  | Heterozygous | NA |  |
|  |  |  |  | c.4855C>T | p.Arg1619* | Nonsense |  | Homozygous | 8.0x 10^-6^ |  |
|  |  |  |  | c.5341G>T | p.Glu1781* | Nonsense |  | Homozygous | NA |  |
|  |  |  |  | c.645G>A | - | Splicing variant | Iran | Homozygous | NA |  |
|  |  |  |  | c.3047T>G | p.Leu1016Arg | Missense. Likelydamaging | Tunis | Homozygous | NA |  |
|  |  |  | Low sperm concentration (12×10^6^/mL)  Very low motility (0.8%)  The CPs or peripheral microtubules were missed in the mid-piece of most flagella; in the principal piece of most flagella, the CPs, peripheral microtubules or ODFs were disorganized; the end piece of most flagella showed a totally disorganized and missing axoneme | c.2675G>A | p.Trp892* | Nonsense | China | Homozygous | NA | 31571197 |
|  |  |  | Low sperm concentration (5.3-15.3×10^6^/mL)  No spermatozoa with progressive motility  The patients with homozygous mutation *CFAP65*(c.5341 G >T) and compound heterozygous mutation *CFAP65*(c.2284C>T and c.1751delC ) exhibited a positive history of chronic respiratory diseases  72.3% and 68.6% had misshapen heads  Unidentifiable or swollen mid pieces and shorter principal pieces of highly irregular width  Flagella were frequently missing, and cytoplasmic residuals were occasionally observed in coil-shaped flagella  Most of the acrosomes were quite thin and showed highly diminished contents with barely recognizable inner and outer acrosomal membranes  A higher incidence of insufficient chromatin condensation  Poorly assembled or missing MS in the midpiece, hyperplasia  of FS  93.3% and 83.9% spermatozoa lacked CP | c.5341 G >T | p.Glu1781* | Nonsense | China | Homozygous | NA | 31413122 |
|  |  |  |  | c.2284C>T | p.Arg762* | Nonsense |  | Heterozygous | 0.000016 |  |
|  |  |  |  | c.1751delC | p.Pro584fs | Frameshift |  | Heterozygous | NA |  |
|  |  |  |  | c.5714_5721del | p.Leu1905fs | Frameshift |  | Heterozygous | NA |  |
|  |  |  |  | c. 3021C>A | p.Asn1007Lys | Missense. |  | Heterozygous | NA |  |
|  |  | *CFAP70* | Low sperm concentration (2, 6.9×10^6^/mL)  Low sperm motility (0,13% )  For patient with mutation of *CFAP70* (c.1723-1G>T), short flagella were most frequently observed  For patient with mutation of *CFAP70* (c.178 T > A), flagella of irregular caliber , abnormal base and acrosome were most frequently observed | c.1723-1G>T | - | Splicing variant | Tunis | Homozygous | NA | 31621862 |
|  |  |  |  | c.178 T > A | p.Phe60Ile | Missense. |  | Homozygous | NA |  |
|  |  | *CFAP74* | Low motility (5,4%) or progressive motility (2,1.7%)  Both exhibited typical symptoms of PCD  Absent, short and irregular caliber flagella were most frequently observed  Lost- or short tail defects and an incomplete MS  MS was disorganized or absent, and the ODFs and peripheral microtubule doublets were disorganized or inadequate | c.983G>A | p.Gly328Asp | Missense | China | Heterozygous | 0.000066 | 32555313 |
|  |  |  |  | c.3532G>A | p.Asp1178Asn | Missense |  | Heterozygous | NA |  |
|  |  |  |  | c.652C>T | p.Arg218Trp | Missense |  | Heterozygous | 0.000091 |  |
|  |  |  |  | c.4331G>C | p.Ser1444Thr | Missense |  | Heterozygous | 0.000082 |  |
|  |  | *BRWD1* | PCD/PCD-likely phenotype  Low sperm concentration (6.21, 8.32×10^6^/mL) except the patient with mutation of *BRWD1* (c. 523 C >T )  Low motility (13.22, 4.21, 7.63%) or progressive motility (4.3, 0.7, 2.1%)  Short flagella were most frequently observed  Absence of IDAs and ODAs  The peripheral microtubule doublets number and the CP were normal | c. 523 C >T | p.His175Tyr | Missense | China | Homozygous | NA | 33389130 |
|  |  |  |  | c. 5573 A >T | p.Gln1858Leu | Missense |  | Homozygous | 0.001095 |  |
|  |  |  |  | c. 1016 T >C | p.Leu339Ser | Missense |  | Heterozygous | NA |  |
|  |  |  |  | c. 166G>A | p.Gly56Ser | Missense |  | Heterozygous | NA |  |
|  |  | STK33 | Low motility (≤40%) or progressive motility (≤20%)  Disorganization in axonemal or peri-axonemal ultrastructures, including abnormal, dispositioned and/or missing peripheral microtubule doublets, inner/outer dynein arms, CP and ODSs | c.1235del | p.Thr412Lys fs*14 | Frameshift | Pakistan | Homozygous | NA | 34155512 |
|  |  | *USP26* | Low progressive motility (26.0, 26.8%)  The rates of coiled flagella and thin heads were obviously higher  Partial defects or loss of the acrosome  A higher rate of disorganization in the axonemal or other peri axonemal structures | c.2473C>G | p.Arg825Gly | Missense | China | Hemizygous | NA | 34202084 |
|  |  |  |  | c.2396A>G | p.Asn799Ser | Missense |  | Hemizygous | NA |  |

**Supplementary Table2. ICSI outcome of MMAF patients reported in the literature**

| **Protein Function** | | **Gene** | **cDNA Variation** | **Amino acid variation** | **ICSI outcome** | | **References PMID** |
| --- | --- | --- | --- | --- | --- | --- | --- |
|  |  |  |  |  | **Fertilization situation** | **ICSI prognosis** |  |
| Flagella structure component related genes | Central pair Microtubules | *SPEF2* | c.12delC | p.Ile4fs | Fertilization | No clinical pregnancy | 31151990 |
|  |  |  | c.1745-2A>G | - |  |  |  |
|  |  | *SPAG6* | c.308C > A | p. Ala103Asp | Fertilization | Pregnancy | 35232447 |
|  |  |  | c. 585delA | p. Lys196Serfs*6 | Fertilization | - |  |
|  | Dynein arms | *DNAH1* | c.4552C>T | p.Gln1518* | Fertilization | Pregnancy | 33929677 |
|  |  |  | c.11787+1G>A | - |  |  |  |
|  |  |  | c.11726_11727delCT | p.Pro3909fs*33 | Fertilization | No clinical pregnancy |  |
|  |  |  | c.12089+1G>A | - |  |  |  |
|  |  |  | c.4552C>T | p.Gln1518* | Fertilization | - |  |
|  |  |  | c.12287G>T | p.Arg4096Leu |  |  |  |
|  |  |  | c.6526-1G>T | - | Fertilization | - |  |
|  |  |  | c.9850G>A | p.Glu3284Lys |  |  |  |
|  |  |  | c.11788-1G>A | p.Gly3930Alafs*120 | Fertilization | Pregnancy | 27094479 |
|  |  |  | c.11788-1G>A | p.Gly3930Alafs*120 | Fertilization | Pregnancy |  |
|  |  |  | c.11788-1G>A | p.Gly3930Alafs*120 | Fertilization | Pregnancy |  |
|  |  |  | c.12796T>C | p.4266Glnext*21 | Fertilization | No clinical pregnancy |  |
|  |  |  | c.5094+1G>A | p.Leu1700Serfs72 | Fertilization | Abortion |  |
|  |  |  | c.11788-1G>A | p.Gly3930Alafs*120 | Fertilization | Pregnancy |  |
|  |  | *DNAH2* | c.2116A>C | p. Lys706Gln | Fertilization | Pregnancy | 33771466 |
|  |  |  | c.11635C>T | p. Arg3879Trp |  |  |  |
|  |  |  | c.5507A>G | p. Lys1836Arg | Fertilization | Pregnancy |  |
|  |  |  | c.9291G>T | p. Glu3097Asp |  |  |  |
|  |  |  | c.4774G>A | p. Glu1592Lys | Fertilization | - |  |
|  |  |  | c.5771G>C | p. Arg1924Pro |  |  |  |
|  |  |  | c.9298C>T | p.Arg3100Trp | Fertilization | Pregnancy | 30811583 |
|  |  |  | c.5770C>T | p.Arg1924Cys | Fertilization | Pregnancy |  |
|  |  |  | c.11500C>T | p.Arg3834* |  |  |  |
|  |  | *DNAH6* | c.6582C>A | p.Asp2194Glu | Fertilization | No clinical pregnancy | 31676830 |
|  |  |  | c.11258G>A | p.Gly3753Asp |  |  |  |
|  |  | *DNAH7* | c.2478dupA | p.V827Sfs*20 | Fertilization | Pregnancy | 35543642 |
|  |  | *DNAH8* | c.6962_6968del | p.His2321Profs*4 | Fertilization | Pregnancy | 32619401 |
|  |  |  | c.2781+1G>T | - | Fertilization | Pregnancy | 33704367 |
|  |  |  | c.10348delT | p.Fhe3459fs |  |  |  |
|  |  | *DNAH10* | c.12838G>A | p.Gly4280Arg | Fertilization | Pregnancy | 34237282 |
|  |  |  | c.5663G>A | p.Arg1888Gln | Fertilization | No clinical pregnancy |  |
|  |  |  | c.11887C>T | p.Arg3963Cys |  |  |  |
|  |  |  | c.12235del | p.Ser4079Alafs*5 | Fertilization | Pregnancy |  |
|  |  |  | c.7260dup | p.Glu2421Argfs*26 |  |  |  |
|  |  | *DNAH17* | c. 4810C>T | p.Arg1604Cys | Fertilization | No clinical pregnancy | 34373205 |
|  |  |  | c.1293_1294del | p.Tyr431* | Fertilization | No clinical pregnancy | 31178125 |
|  |  |  | c .7994_8012del | p.Gly2665-Glufs* |  |  |  |
|  |  |  | c.5486G>A | p.Cys1829Tyr | Fertilization | No clinical pregnancy |  |
|  |  |  | c.5486G>A | p.Cys1829Tyr | Fertilization | No clinical pregnancy |  |
|  |  |  | c.[10496C>T;10784T>C] | [Pro3499Leu;Leu3595Pro] | Fertilization | No clinical pregnancy |  |
|  |  |  | c.10486_10497dup | p.Val3496_Pro3499dup | Fertilization | Pregnancy |  |
|  |  |  | c.12915+1C > T | - | Fertilization | Pregnancy | 33423959 |
|  |  |  | c.13202G > A | p.Pro4401Leu |  |  |  |
|  |  |  | c.8512-2A > G | - | Fertilization | Pregnancy |  |
|  |  |  | c.13294C > T | p.Arg4432Cys |  |  |  |
|  |  |  | c.1048 C > T | p.Arg350* | No fertilization | No clinical pregnancy | 3412683 |
|  |  |  | c.3390G > A | p.Met1130Ile |  |  |  |
|  |  | *CFAP43* | c.3661-2A>-(delA) | - | Fertilization | Pregnancy | 30251428 |
|  |  | *CFAP44* | c.2935_2944del: | p. Asp979* | Fertilization | No clinical pregnancy |  |
|  |  |  | c. 1769 T >A: | p.Leu590Gln | Fertilization | No clinical pregnancy |  |
|  |  |  | c.2005_2006del: | p.Met669Val fs*13 | Fertilization | Pregnancy |  |
|  |  |  | c. 3262 G >A: | p.Gly1088Ser | Fertilization | Pregnancy |  |
|  |  |  | c. 1718 C >A | p.Pro573His |  |  |  |
|  |  | *CCDC39* | c.983 T>C | p. Leu328Pro | Fertilization | Pregnancy | 34674941 |
|  | Radial Spoke | *CFAP61* | c.1654C>T | p.Arg552Cys | Fertilization | - | 35387802 |
|  |  |  | c.2911G>A | p.Asp971Asn |  |  |  |
|  |  |  | c.144–2A>G | - | Fertilization | Pregnancy |  |
|  |  |  | c.1666G>A | p.Gly556Arg |  |  |  |
|  |  | *CFAP251* | c.1192-3C>G | - | Fertilization | Pregnancy | 35087568 |
|  |  | *CFAP206* | c.1430dupA; | p.Asn477Lysfs*15 | Fertilization | No clinical pregnancy | 34255152 |
|  | N-DRC | *DRC1* | c. 1660C>T | p.Arg554* | Fertilization | Pregnancy | 34169321 |
|  | ODF | *ODF2* | c.242 A>G | p.Lys81Arg | Fertilization | Pregnancy | 35102900 |
|  |  | *CFAP58* | c.323C>T | p.Ser108Leu | Fertilization | Pregnancy | 33314088 |
|  |  |  | c.1855C>T | p.Arg619Trp |  |  |  |
|  | FS | *FSIP2* | c.16246_16247insCCCAAATATCACC | p. Thr5416fs*7 | Fertilization | No clinical pregnancy | 33631238 |
|  |  |  | c.17323C > T | p.Gln5774* |  |  |  |
|  |  |  | c.1494C > A | p.Cys498* | Fertilization | Pregnancy | 35672654 |
|  |  |  | c.11020_11024del | p.Tyr3675Cysfs*3 |  |  |  |
|  |  |  | c.1750T>A | p.Cys584Ser | Fertilization | Pregnancy | 34935173 |
|  |  |  | c.13600A>G | p.Ile4534Val |  |  |  |
|  |  |  | c.1750T>A | p.Cys584Ser | Fertilization | Pregnancy |  |
|  |  |  | c.13600A>G | p.Ile4534Val |  |  |  |
|  |  | *AKAP3* | c.2286_2287del | p.His762Glnfs*22 | Fertilization | No clinical pregnancy | 35228300 |
|  |  |  | c.44G>A | p.Cys15Tyr | Fertilization | Pregnancy |  |
|  |  | *AKAP4* | c. 1285 C >T | p.Arg429Cys | Fertilization | Pregnancy | 34415320 |
|  |  |  | c.1285 C >T | p.Arg429Cys | Fertilization | Pregnancy |  |
|  |  |  | c. 1285 C >T | p.Arg429Cys | Fertilization | No clinical pregnancy |  |
|  | Centrosome | *CEP135* | c. 1364 A >T | p.Asp455Val | Fertilization | No clinical pregnancy | 28866084 |
| Genes encoding proteins interacting with other proteins to participate in flagellar assembly | | *TTC21A* | c.3450+2delT | - | Fertilization | No clinical pregnancy | 35920310 |
|  |  | *TTC29* | c.1107C>G | p.Tyr369* | Fertilization | Pregnancy | 31735294 |
|  |  |  | c.412_425del | p.Asp138Leufs*10 | Fertilization | No clinical pregnancy |  |
|  |  |  | c.1107C>G | p.Tyr369* | Fertilization | Pregnancy |  |
|  |  |  | c.977+1G>T | p.Ser326Profs*8 |  |  |  |
|  |  | *IFT44/WDR19* | c. 3811 A >G | p.Lys1271Gln | Fertilization | Pregnancy | 32323121 |
|  |  | *ARMC2* | c.1264C > T | p.Arg422* | Fertilization | Pregnancy | 35543806 |
|  |  | *CCDC34* | c.731dup | p.Asn244Lysfs*3 | Fertilization | Pregnancy | 34348960 |
|  |  | *DNHD1/CCDC35* | c.5560C>T | p.Arg1854Cys | Fertilization | No clinical pregnancy | 34932939 |
|  |  |  | c.6498T>G | p.Tyr2166∗ |  |  |  |
|  |  |  | c.8782C>T | p.Arg2928∗ | Fertilization | Pregnancy |  |
|  |  |  | c.522_525del | p.Arg174Serfs∗3 | Fertilization | Pregnancy |  |
|  |  |  | c.911G>A | p.Arg304Gln |  |  |  |
|  |  |  | c.425_428del | p.Asp142Valfs∗35 | Fertilization | No clinical pregnancy |  |
|  |  |  | c.4072C>T | p.Arg1358Cys |  |  |  |
|  |  |  | c.8909A>G | p.Tyr2970Cys | Fertilization | No clinical pregnancy |  |
|  |  |  | c. 9649C>T | p.Arg3217∗ | Fertilization | Pregnancy |  |
|  |  |  | c.12473A>G | p.His4158Arg |  |  |  |
|  |  |  | c.14234T>C | p.Val4745Ala | Fertilization | Pregnancy |  |
|  |  |  | c.5347delC | p.Gln1783Serfs∗28 |  |  |  |
| Genes of which encoding proteins’location and function were undefinedor questionable | | *AK7* | c.1846G > A | p.Glu616Lys | No fertilization | - | 34854019 |
|  |  | *CFAP47* | c.7154T>A | p.Ile2385Asn | No fertilization | - | 33472045 |
|  |  |  | c.5224A>G | p.Ser1742Gly | Fertilization | Pregnancy |  |
|  |  |  | c.8668C>A | p.Pro2890Thr | Fertilization | Pregnancy |  |
|  |  |  | GRCh37/hg19.Xp21.1x0 | eliminating the entire CFAP47 | Fertilization | Pregnancy |  |
|  |  | *CFAP65* | c.5341G>T | p.Glu1781* | Fertilization | No clinical pregnancy | 31413122 |
|  |  |  | c.2284C>T | p.Arg762* | Fertilization | No clinical pregnancy |  |
|  |  |  | c.1751delC | p.Pro584fs |  |  |  |
|  |  |  | c.5714_5721del | p.Leu1905fs | Fertilization | No clinical pregnancy |  |
|  |  |  | c.3021C>A | p.Asn1007Lys |  |  |  |
|  |  | *CFAP70* | c.1723-1G>T | - | Fertilization | Pregnancy | 31621862 |
|  |  | *CFAP74* | c.983G>A | p.Gly328Asp | Fertilization | Pregnancy | 32555313 |
|  |  |  | c.3532G>A | p.Asp1178Asn |  |  |  |
|  |  | *USP26* | c.2473C>G | p.Arg825Gly | Fertilization | Pregnancy | 34202084 |
|  |  |  | c.2396A>G | p.Asn799Ser | Fertilization | Pregnancy |  |
